# Plant Acquisitive Strategies Promote Resistance and Temporal Stability of Semiarid Grasslands

**DOI:** 10.1111/ele.70110

**Published:** 2025-04-03

**Authors:** Pu Yan, Nianpeng He, Marcos Fernández‐Martínez, Xian Yang, Yiping Zuo, Hao Zhang, Jing Wang, Shiping Chen, Jian Song, Guoyong Li, Enrique Valencia, Shiqiang Wan, Lin Jiang

**Affiliations:** ^1^ School of Biological Sciences Georgia Institute of Technology Atlanta Georgia USA; ^2^ Key Laboratory of Sustainable Forest Ecosystem Management–Ministry of Education Northeast Forestry University Harbin China; ^3^ CREAF Cerdanyola del Vallès, Barcelona Spain; ^4^ School of Ecology Sun Yat‐Sen University Guangzhou China; ^5^ Key Laboratory of Ecosystem Network Observation and Modeling, Institute of Geographic Sciences and Natural Resources Research Chinese Academy of Sciences Beijing China; ^6^ State Key Laboratory of Vegetation and Environmental Change, Institute of Botany Chinese Academy of Sciences Beijing China; ^7^ School of Life Sciences, Institute of Life Science and Green Development Hebei University Baoding China; ^8^ International Joint Research Laboratory for Global Change Ecology, School of Life Sciences Henan University Kaifeng China; ^9^ Department of Biodiversity, Ecology and Evolution, Faculty of Biological Science Complutense University of Madrid Madrid Spain

**Keywords:** acquisitive strategies, conservative strategies, drought, plant functional traits, semi‐arid grasslands, stability

## Abstract

Among ecologists, it is widely believed that conservative growth strategies of plants are crucial for sustaining ecosystem stability, while the potential stabilising role of acquisitive strategies has received little attention. We investigated the relationships between plant traits and three stability dimensions—temporal stability, resistance and resilience—using two complementary datasets from drought‐affected semi‐arid grasslands: a temporal plant community survey from a single site and a 1000‐km transect survey with satellite‐derived productivity estimates. We found strikingly consistent patterns from the two datasets, with grasslands dominated by acquisitive strategies exhibiting greater resistance and temporal stability of productivity. Acquisitive strategies enhance stability by facilitating drought escape and avoidance, rather than drought tolerance typically associated with conservative strategies. These results highlight the important but underappreciated role of acquisitive strategies in enhancing ecosystem resistance to disturbances and maintaining temporal stability in semi‐arid grasslands.

## Graphical abstract

Plant economic traits are important in regulating multidimensional stability of the productivity of semi‐arid grasslands in China. Temporal stability, resistance and resilience are higher in grasslands characterised by more acquisitive strategies.

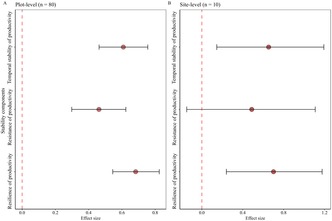

## 1 Introduction

Understanding mechanisms regulating the stability of natural ecosystems has been a central focus of ecological research for decades (McNaughton 1977; Pimm 1984; Ives and Carpenter 2007; de Bello et al. 2021), in part because of the need to safeguard ecosystem products and services in an era where the Earth is undergoing significant environmental change (Costanza et al. 2014; Isbell et al. 2017; Watson et al. 2019). An important component of ongoing global environmental change is the increasing frequency of extreme climatic events, which can have substantial impacts on the structure and functioning of natural communities (Smith 2011; Smith et al. 2024). For example, recent global‐scale studies reveal that intensified drought events driven by climate change are causing biomass losses far exceeding expectations (Smith et al. 2024). These biomass losses often result from disruptions to species composition and plant productivity (Hoover et al. 2014; Sun et al. 2022), with potentially profound implications for the compositional and functional stability of impacted ecosystems (Xu, Yang, et al. 2022).

Much of the research on stability has traditionally focused on a single dimension, particularly temporal stability (the degree of invariability over time) (Tilman 1999; Craven et al. 2018; García‐Palacios et al. 2018). However, ecologists have increasingly recognised the importance of studying ecological stability as a multidimensional concept (Donohue et al. 2013, 2016; Xu, Yang, et al. 2022; Chen et al. 2023), as different stability dimensions may capture different aspects of dynamic properties of an ecological system. For example, resistance reflects the ability of a system to resist displacement from perturbation (Pimm 1984; Donohue et al. 2013; Xu, Yang, et al. 2022), whereas resilience captures the ability of the system to return to its original state after perturbation (Pimm 1984; Ives and Carpenter 2007; Pimm et al. 2019; Xu, Yang, et al. 2022). Together, temporal stability, resistance and resilience provide a more comprehensive characterisation of how ecological systems maintain their properties in the face of disturbances (Pimm 1984; Eisenhauer et al. 2024). Recent research has shown that these stability dimensions may covary. For instance, resistance has been found to be positively associated with temporal stability (Isbell et al. 2015), and negatively associated with resilience (Chen et al. 2021). These relationships, however, may vary substantially across environmental context (Radchuk et al. 2019). Despite these insights, ecological drivers of these stability dimensions remain poorly understood, limiting our ability to predict ecosystem responses to climate extremes and other disturbances.

**FIGURE 1:**
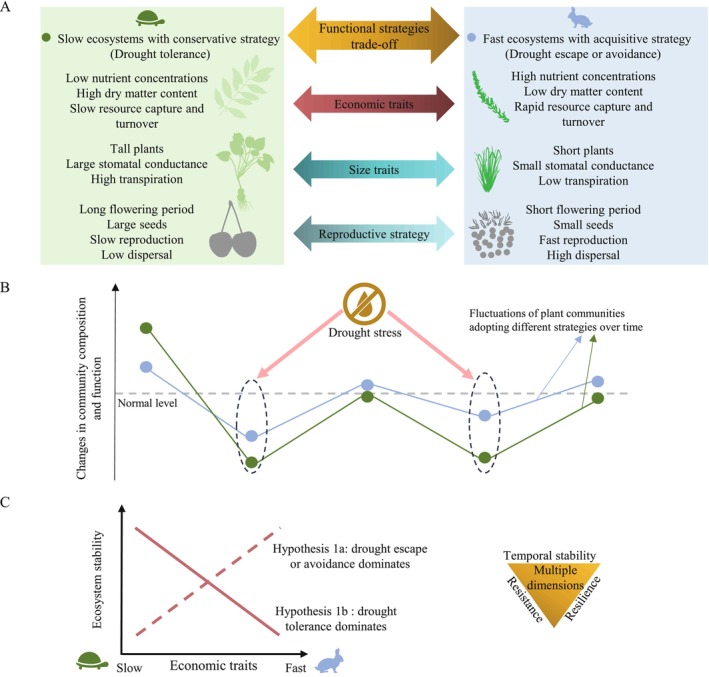
Schematic diagrams illustrating the trade‐off between plant growth strategies and their role in driving multidimensional ecological stability. Panel (A) highlights how plant growth strategy trade‐offs are reflected in functional traits, with fast‐growing species exhibiting acquisitive traits (e.g., high SLA and low LDMC) and slow‐growing species characterised by conservative traits (e.g., low SLA and high LDMC). Panel (B) provides a hypothetical example of how drought stress in water‐limited environments (e.g., semi‐arid grasslands) impacts the composition and function (e.g., productivity) of plant communities dominated by these contrasting growth strategies, with slow‐growing species associated with higher drought tolerance and fast‐growing species linked to greater drought avoidance or escape strategies. Panel (C) depicts alternative hypotheses of how plant growth strategies influence ecosystem stability components, particularly temporal stability and resistance, which are key indicators of an ecosystem's ability to withstand disturbances and maintain stability over time. Traditionally, conservative strategies are considered positively linked to these stability components (solid lines), while the role of acquisitive strategies, which may in principle also contribute to stability (dashed lines), remains underexplored.

Advances in trait‐based ecology have highlighted the utility of functional traits in delineating plant life history strategies (Wright et al. 2004; Reich 2014; Díaz et al. 2016; Bergmann et al. 2020; Carmona et al. 2021). A prominent example of the progresses made is the identification of the plant economics spectrum, which characterises species with fast growth rate and rapid resource uptake (i.e., acquisitive growth strategies) at one end of the spectrum and slow‐growing species with long life spans (i.e., conservative growth strategies) at the other end of the spectrum (Wright et al. 2004; Reich 2014). Acquisitive species may quickly exploit available resources following disturbance, conferring greater resilience to communities dominated by such species (Craven et al. 2018; de Bello et al. 2021). On the other hand, species with conservative strategies are generally thought to be more tolerant of climate extremes and disturbances due to their ability to invest in durable tissues, store resources during favourable periods, and maintain relatively low metabolic rates, such that communities dominated by conservative species are expected to be more resistant to disturbances and more temporally stable (Craven et al. 2018; de Bello et al. 2021). While this hypothesis has received considerable empirical support (Polley et al. 2013; Craven et al. 2018; Wilcox et al. 2021; Hou et al. 2022; Valerio et al. 2022; Xu, Li, et al. 2022; Luo et al. 2023), it notably does not account for the fact that plants, such as herbaceous species in grasslands, can respond to disturbances via alternative mechanisms. For example, herbaceous plants are known to respond to drought via drought tolerance (the ability to withstand dehydration; e.g., via enhanced carbohydrate storage), drought escape (the ability to grow and reproduce rapidly before the onset of severe drought; e.g., via earlier flowering), or drought avoidance (the ability to increase water use efficiency and avoid dehydration; e.g., via low stomatal conductance) (Ludlow 1989; Kooyers 2015; Bristiel et al. 2019). It then follows that plants and ecosystems may mitigate mortality and functional loss in the event of drought via different drought response mechanisms, such that conservative plant growth strategies, which are often associated with high drought tolerance, may not be the only pathway to high ecosystem stability (Figure 1). This possibility, however, has not been assessed.

In this study, we explored the linkage between plant functional traits and multidimensional ecosystem stability, including temporal stability, resistance and resilience, in semi‐arid grasslands of Northern China. Semi‐arid grasslands cover a substantial fraction of the Earth's terrestrial area and exhibit strong responses to variation in water availability (Currier and Sala 2022), providing excellent opportunities for studying the stability of natural ecosystems under drought influences. We used two complementary datasets (Figure S1; Table S1) to investigate trait–stability relationships: a 6‐year (2005–2010) dataset from a single site, which comprised temporal data on species composition and productivity measured in situ, and a 1000‐km grassland transect survey dataset, which contained snapshot data on species composition coupled with long‐term satellite‐based temporal productivity estimates (2000–2022). Both datasets feature in situ measurements of leaf and root traits (Table S2). We examined the three dimensions of stability for both species composition (i.e., compositional stability) and productivity (i.e., functional stability) for the temporal community survey data; for grassland transect data, we focused on the stability of productivity (functional stability) as temporal compositional data were not available. Collectively, these two independent datasets enabled us to assess the generality and consistency of trait–stability relationships across diverse environmental contexts. Our study aimed to answer one main question: How do plant functional traits, particularly those reflecting acquisitive and conservative strategies, influence multidimensional stability?

## 2 Methods

### 2.1 Study Region

Two independent sets of plant community survey data, coupled with in‐situ measured trait data, were collected from the semi‐arid grasslands of the Mongolian plateau (Figure S1; Table S1). These natural grasslands, with minimal anthropogenic disturbance (e.g., livestock grazing, cropping), were selected to assess plant community dynamics and trait–stability relationships under natural ecological conditions. The region experiences a temperate monsoon climate, with precipitation primarily concentrated during the growing season (from early May to late October). Water is recognised as the most important limiting resource in these semi‐arid grasslands (Bai et al. 2008; Sun et al. 2022).

The first dataset, collected from a field experiment at a single site, contained 6‐year annual data on both species composition and productivity. The second dataset, collected from a grassland transect survey, lacked temporal species composition data but contained longer‐term (2000–2022) productivity data estimated via remote sensing. These two datasets are complementary in that the temporal community dynamics data from the single experimental site permit in‐depth analyses of both compositional and productivity stability, albeit over a relatively short timescale, while the multi‐site survey data allow the assessment of grassland productivity stability over longer timescales across localities differing in climatic conditions (Table S1). Together, these two datasets provide a more robust assessment of trait–stability relationships in semi‐arid grassland ecosystems of China. In total, the data contained 316 species and 22 plant leaf and root traits (Table S2). We classified all plant species encountered into annuals and perennials, based on the plant life‐cycle database provided by Poppenwimer et al. (2023).

### 2.2 Temporal Plant Community Data

This dataset originates from the control plots of a randomised complete block field experiment initiated in 2005 (Xu, Yang, et al. 2022). The experiment was conducted at the Duolun Restoration Ecology Research Station (42°02′ N, 116°17′ E) on the southern Inner Mongolian Plateau, China, where the average annual precipitation is 378 mm and the average annual temperature is 2.1°C. Four blocks (92 × 60 m) were established, separated by 5‐m‐wide buffer zones. Each block contained one control plot (44 × 28 m), along with other treatment plots (also 44 × 28 m each). In each plot, two 6 × 6 m subplots were positioned in two corners. Within each of the 6 × 6 m subplots, a single 1 × 1 m permanent quadrat, placed at the centre of the subplot, was used for vegetation survey.

Plant communities in all plots were surveyed annually from 2005 to 2010 in August, coinciding with the peak biomass of the study grassland. Plant cover, serving as a reasonable proxy for primary productivity in the study grassland (Xia et al. 2009; Xu, Yang, et al. 2022), was estimated in lieu of biomass to avoid disturbances associated with destructive biomass estimation. Plant cover was assessed using the point intercept method, by placing a 1 × 1 m frame containing 100 10 × 10 cm grids positioned over the 1 × 1 m permanent quadrat. Each species within each grid was identified, and the percentage cover of each species was estimated based on its presence within the 100 grids. The percentages were summed across all species within each quadrat, and then averaged across the two subplots to obtain the total community cover for each plot.

### 2.3 Grassland Transect Survey Data

A field survey was conducted during the peak growing season of the semi‐arid grasslands in Inner Mongolia from July to August 2018. Ten undisturbed sites were established along an east–west precipitation gradient spanning 1000 km, with distances of over 80 km between adjacent sites (Figure S1). Across sites, the annual average temperature ranged from 0.1°C to 5.8°C, and precipitation ranged between 183 and 425 mm (Table S1). At each site, we established eight plots (1 × 1 m each) within a relatively homogenous area spanning 1000 × 1000 m. We identified all species within each plot, recorded species richness and height and finally harvested the aboveground materials. All harvested samples were dried at 85°C for 72 h and weighed to obtain the aboveground dry biomass.

### 2.4 Trait Measurement

#### 2.4.1 Temporal Plant Community Data

We quantified 17 plant morphological and chemical traits, including plant height (height), leaf life span (LLS), leaf area (LA), leaf dry matter content (LDMC), leaf nitrogen concentration (LNC), root nitrogen concentration (RNC), specific leaf area (SLA), specific root area (SRA), specific root length (SRL), root dry matter content (RDMC), fine root length and diameter, total root depth and length and root base diameter. These traits capture key aspects of plant functional strategies (Figure S2). Additionally, seed dry mass and flowering duration were also measured and recorded. All trait measurements follow standardised protocols (Cornelissen et al. 2003; Perez‐Harguindeguy et al. 2016); specific details on trait measurements are provided in Supporting Information (Text S1).

#### 2.4.2 Grassland Transect Survey

To quantify plant functional strategies (Figure S2), we measured 11 morphological and chemical traits of 284 plant species, including height, LA, nitrogen and phosphorus concentrations in leaves and roots (LNC, leaf phosphorus concentration [LPC], RNC and root phosphorus concentration [RPC]), SLA, LDMC and RDMC. In addition, stomatal area and conductance (1/Δ^18^O) were also measured. For further details regarding plant trait measurement, see Text S1, and other sources published (Wang and Wen 2022; Yan, Fernández‐Martínez, et al. 2023; Yan, He, et al. 2023).

### 2.5 Identifying Drought Events

For the Duolun experimental site, growing season precipitation in 2007 and 2009 fell below the 5th percentile of the historical precipitation distribution (1953–2010), whereas precipitation in other study years was between 5th and 95th percentiles of the distribution (Figure S3). Consequently, 2007 and 2009 were classified as drought years, and the remaining study years were considered typical‐climatic years.

For the grassland transect sites, we quantified drought events between 2000 and 2022 for each site using the standardised precipitation–evapotranspiration index (SPEI, https://spei.csic.es/). Typical‐climatic years correspond to SPEI values between −0.67 and 0.67 (Vicente‐Serrano et al. 2010), and drought years were identified if the SPEI values fell below −0.67. While these thresholds provide a practical baseline for identifying climatic conditions, we acknowledge that global change factors, such as increased climate variability and shifting baselines, may alter the definition of typical/atypical climate conditions over time. This underscores the need to carefully consider reference states when studying ecosystem stability in the context of global changes. A drought year used to calculate resistance or resilience also needs to meet two additional criteria: (1) The year preceding drought must be either a drought or typical‐climatic year (i.e., not alternating between wet and dry); (2) A drought year used to calculate resilience must be followed by a typical‐climatic year (Chen et al. 2023). Detailed climatic conditions for each year at each site, along with the summary table used to distinguish three consecutive years for calculating resistance and resilience, are provided in the Supporting Information (Figure S4; Table S3).

### 2.6 Stability Metrics

For data collected at the Duolun station over consecutive years (2005–2010), we calculated temporal stability, resistance and resilience for both species composition (i.e., compositional stability) and productivity (i.e., productivity stability). For grassland transect sites, we focused on temporal stability, resistance and resilience of productivity as temporal compositional data were not available. We focused on the stability of productivity not only because productivity is a widely used and easily measurable metric of ecosystem functioning, but also because it is a key function representing the energy available to higher trophic levels. In addition, ecosystem productivity is known to respond readily to climate extremes, particularly drought (Knapp and Smith 2001; Smith et al. 2024; Yu et al. 2025), making it imperative to better understand mechanisms regulating its stability. Examining productivity also allows for comparisons between our findings and those of previous studies on ecological stability, which have mainly focused on productivity stability (García‐Palacios et al. 2018; Liu et al. 2022). We used EVI (Enhanced Vegetation Index) from the MODIS satellite imagery MOD13Q1 product (250  × 250 m, 2000–2022 years) as the proxy of aboveground biomass (i.e., productivity) along the transect. The EVI metric is a reliable indicator of above‐ground plant biomass along the transect (*r* = 0.87; Figure S5). Data based on normalised difference vegetation index (NDVI) produced similar results (Figure S6).

We quantified temporal stability of productivity as the following equation:

(1) $$\text{Temporal stability}=\frac{\mu }{\sigma }$$

where *μ* is the mean ecosystem productivity and *σ* is the standard deviation in ecosystem productivity across all years.

We quantified resistance of productivity as the following equation:

(2) $$\text{Resistance}=\ln\frac{\;\;P_{\text{drought}}}{\;P_{\text{typical}-\text{climatic}}}$$

where $P_{\text{typical}-\text{climatic}}$ is the ecosystem productivity averaged across all typical‐climatic growing seasons. At the Duolun Station experimental site, these typical‐climatic years are 2005, 2006, 2008 and 2010. For the grassland transect sites, ‘typical‐climatic’ corresponds to seasons with SPEI values between −0.67 and 0.67 (Isbell et al. 2015; Chen et al. 2023). *P*_drought_ represents ecosystem productivity in drought years, defined by SPEI values below −0.67 for the grassland transect sites.

We quantified resilience of productivity as the following equation:

(3) $$\text{Resilience}=\ln\frac{\;\;P_{\text{post}-\text{drought}}}{\;P_{\text{drought}}}$$

where $P_{\text{post}-\text{drought}}$ represents ecosystem productivity in the year following the drought.

We further compared resistance and resilience values with those calculated using two alternative methods (Isbell et al. 2015; Liu et al. 2022). The results based on different methods were generally consistent with each other (*r* > 0.7; Figure S7).

For data collected at the Duolun station, we calculated compositional stability dimensions using the Bray–Curtis dissimilarity metric based on plant cover data, with the ‘beta.multi.abund’ function from the R package ‘betapart’ (Baselga and Orme 2012). Here, the Bray–Curtis dissimilarity metric quantified the compositional dissimilarity between two consecutive samples across time. The metric ranges from 0 (identical composition) to 1 (completely different composition). Compositional temporal stability (across all years) and resistance (drought vs. pre‐drought years) were quantified as 1—dissimilarity, while compositional resilience was defined as the dissimilarity between drought and post‐drought communities (Xu, Yang, et al. 2022).

### 2.7 Community Weighted Mean Traits

We used the ‘dbFD’ function of the ‘vegan’ R package to calculate the community weighted mean (CWM) of each trait, representing the community‐level trait value weighted by species abundance. To assess community‐level drought‐induced trait shifts at the Duolun site, we calculated the relative change in trait CWM between drought years (2007 and 2009, CWM_drought_) and typical‐climatic years (CWM_typical‐climatic_), expressed as a fraction of CWM_typical‐climatic_.

### 2.8 Statistical Analyses

All statistical analyses were completed in R (R version 4.1.0; R Development Core Team 2021). Given the primary focus of this study on the relationships between plant traits and multidimensional ecological stability, we adhered to recent recommendations for good research practises by emphasising effect sizes with confidence intervals (Popovic et al. 2024), instead of relying solely on null hypothesis‐based statistical significance (i.e., *p*‐values).

### 2.9 Changes in Community Functional Composition Induced by Drought

To assess the statistical significance of drought‐induced changes in the functional composition of plant communities at the Duolun site, we constructed 95% confidence intervals for the relative changes in trait CWMs using a bootstrapping approach. Specifically, we resampled the observed CWM values for drought years (2007 and 2009, CWM_drought_) and typical‐climatic years (CWM_typical‐climatic_) 999 times. This analysis was conducted using the ‘boot’ and ‘boot.ci’ functions from the R package ‘boot’.

### 2.10 Relationships Between Plant Traits and Multidimensional Stability

We first examined the associations between individual functional traits and the three stability dimensions (temporal stability, resistance and resilience) using temporal plant community data. Traits that did not show significant relationships with any stability dimensions during exploratory data analysis were excluded from further analyses. To streamline the analysis, we performed principal component analysis (PCA) on key economic and size traits following Craven et al. (2018). Economic traits included LLS, SLA, LDMC, RDMC and SRA, while size traits included plant height, root depth, root length and fine root length. The first principal component (PC1) from each PCA was extracted to represent economic and size traits, respectively (Figures S2, S8 and S9), and later related to stability components. For stability dimensions that were significantly associated with only a few trait variables (< 3), we directly selected the best trait predictor variables. We then constructed regression models with six different stability dimensions (temporal stability of productivity, resistance of productivity, resilience of productivity, temporal stability of composition, resistance of composition and resilience of composition) as response variables and the two sets of traits (economic and size traits) as explanatory variables.

Likewise, we used multi‐site data from the transect survey to evaluate the relationships between plant traits and the three dimensions of productivity stability. While linear mixed‐effects models are generally appropriate for handling hierarchically structured data (i.e., plots nested within each survey site), all plots within a given survey site in our study actually shared the same stability values for all three stability dimensions. This is because all the stability values were derived from remotely sensed EVI data, which do not have sufficient spatial resolution within a site for us to extract unique EVI values for individual plots. As such, linear mixed‐effects models cannot be performed. Therefore, we resorted to Bayesian regression models relating the three dimensions of productivity stability to plant traits. We first ran regressions at the plot level. Given that plot‐level data within a given site are not completely independent from each other, we also conducted regressions using data averaged at the site level, treating each site as a single observation. We conducted Bayesian partial regression analysis to explore the association between economic traits and temporal stability, resistance and resilience of productivity, while controlling for the potential effects of growing season precipitation. Growing‐season precipitation was log‐transformed prior to this analysis. We also used random forest modelling, a machine learning approach for effectively dealing with collinearity problems (Breiman 2001), to identify the primary drivers of ecosystem stability.

We employed Bayesian estimation for all model fittings, utilising two Markov chain Monte Carlo chains, 3000 iterations with 1000 as warm‐up, and weakly informative priors (mean = 0 and standard deviation = 10) (Umaña et al. 2023). The Bayesian models were created in the Stan computational framework (http://mc–stan.org/) accessed with the ‘brms’ package (Bürkner 2017). To gauge model convergence, we conducted a visual inspection of trace plots and computed $\hat{R}$ values (the ratio of effective sample size to the total number of iterations). All $\hat{R}$ values were below 1.01. All variables were standardised (mean = 0, standard deviation = 1) prior to analysis. Model verification and comparison were performed using LOOIC (leave‐one‐out cross‐validation information criterion) and ELPD (expected log predictive density) metrics, assessed with the ‘loo’ package. Posterior prediction checks were performed using the ‘bayesplot’ package (Gabry et al. 2019). The Pareto shape parameter (*K* values) was used to identify anomalous observations.

## 3 Results

**FIGURE 2:**
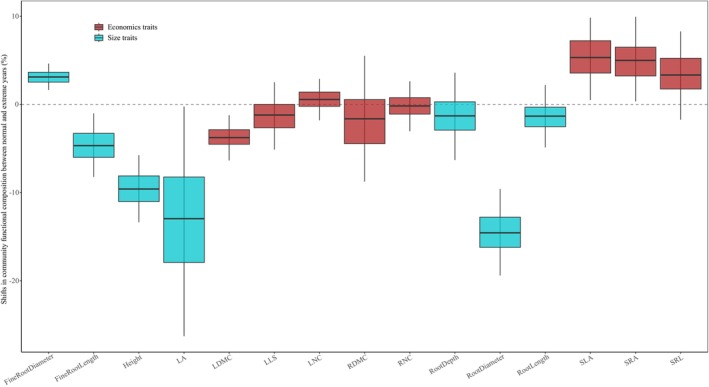
Drought‐induced changes in community‐weighted means of plant functional traits at the Duolun site. The boxplot is customised in such a way that the thick central line represents the mean percent shift in community‐weighted mean traits relative to non‐drought years, with the boxes indicating the interquartile range and the whiskers extending to the bootstrapped 95% confidence intervals. FineRootDiameter, fine root diameter; FineRootLength, fine root length; Height, plant height; LA, leaf area; LDMC, leaf dry mass content; LLS, leaf life span; LNC, leaf nitrogen concentration; RDMC, root dry mass content; RNC, root nitrogen concentration; RootDepth, root depth; RootDiameter, root diameter; RootLength, root length; SLA, specific leaf area; SRA, specific root area; SRL, specific root length.

We observed significant shifts in the trait CWMs of Duolun grasslands between typical‐climatic and drought years (Figure 2). Specifically, during drought years, grasslands shifted towards traits associated with acquisitive growth strategies, characterised by larger SLA and SRA, and lower LDMC values (Figure 2). In addition, plant size declined under drought (reduced LA and height; Figure 2). Correspondingly, the proportion of annual plants increased during drought years (2007 and 2009; Figure S10).

**FIGURE 3:**
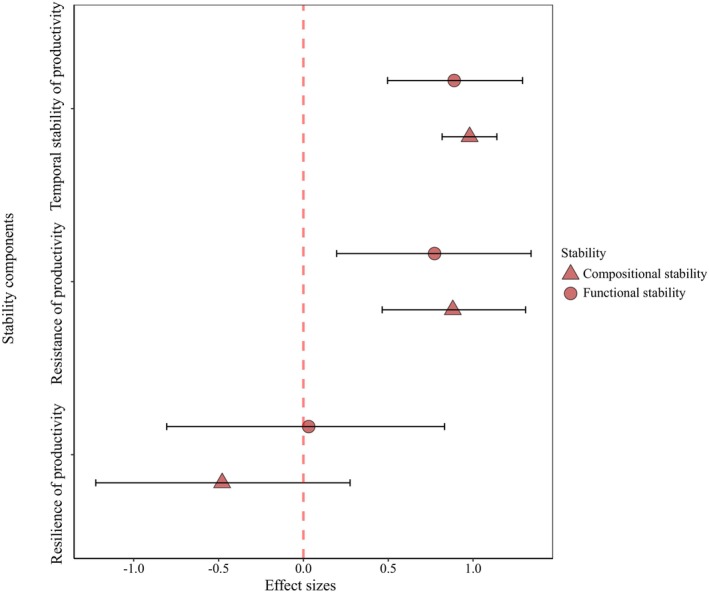
The effects of economic traits on functional and compositional temporal stability, resistance and resilience at the Duolun site. Circles represent mean standardised effect sizes with 95% credible intervals (error bars), derived from Bayesian models. Functional stability refers to the stability of productivity, while compositional stability refers to the stability of species composition. Economic traits capture the acquisition–conservative strategy trade‐offs, with higher values denoting acquisitive strategies and lower values indicating conservative strategies.

The analysis of temporal data from the Duolun site showed that CWMs of economic traits (larger values indicative of more acquisitive strategies) exhibited positive relationships with productivity and compositional temporal stability and resistance, but weak and nonsignificant relationships with productivity and compositional resilience (Figure 3; Table S4). By contrast, CWMs of size traits (larger values indicative of larger plants) exhibited negative relationships with productivity and compositional temporal stability, as well as with compositional resistance, and positive relationships with productivity and compositional resilience (Table S4). The identities of specific traits driving these patterns varied between productivity temporal stability and resistance, but were more constant for their corresponding compositional metrics (Figure S11; Tables S5 and S6).

**FIGURE 4:**
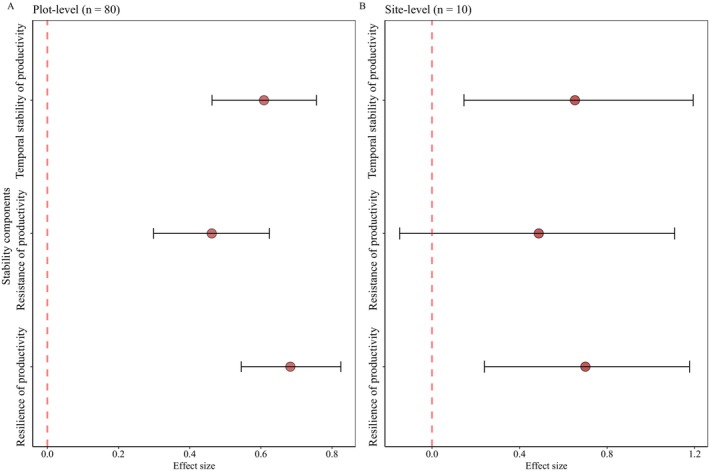
The effects of economic traits on the stability of productivity, including temporal stability, resistance and resilience, based on grassland transect survey data. Panels (A) and (B) show results at the plot level (*n* = 80) and site level (*n* = 10), respectively. Circles represent mean standardised effect sizes with 95% credible intervals (error bars), derived from Bayesian models. Economic traits reflect acquisition–conservative strategy trade‐offs, with higher values denoting acquisitive strategies and lower values representing conservative strategies. Compositional stability metrics cannot be calculated for this transect survey dataset due to the lack of temporal community composition data.

The analysis of grassland transect data showed that CWMs of economic traits exhibited statistically significant, positive associations with productivity temporal stability (*β* = 0.61 [0.46, 0.75]), resistance (*β* = 0.46 [0.30, 0.63]) and resilience (*β* = 0.68 [0.54, 0.82]); qualitatively similar results were obtained using data averaged at the site level (Figure 4; Figure S12; Table S7). CWMs of size traits exhibited significant negative relationships solely with resistance (*β* = −0.34 [−0.52, −0.16]; Table S7). Focusing on individual traits, temporal stability, resistance and resilience of productivity all increased as SLA, LNC, LPC, RNC, RPC increased, but decreased as LDMC increased (Figure S12; Table S8). Resistance, however, decreased as LA and height increased. The positive relationships between economic traits and productivity temporal stability (*R* ^2^ = 0.39 [0.24, 0.50]), resistance (*R* ^2^ = 0.11 [0.014, 0.24]) and resilience (*R* ^2^ = 0.48 [0.35, 0.58]) remained after accounting for the effect of growing season precipitation (Figure S13). Random forest analysis further identified the importance of economic traits for productivity temporal stability, resistance and resilience (Figure S14).

## 4 Discussion

Contrary to the prevailing belief that plant conservative growth strategies confer ecosystem stability (Craven et al. 2018; de Bello et al. 2021), we found that semi‐arid grasslands dominated by more acquisitive growth strategies exhibit greater resistance and temporal stability. This result, previously unreported to our knowledge, is robust across both a multi‐year plant community survey at a single site and a large‐scale transect survey coupled with satellite‐derived temporal productivity data. This consistent pattern between two independent datasets underscores the critical role of acquisitive strategies in maintaining productivity stability of our study semi‐arid grasslands. This result, however, is not completely surprising, as plant communities are known to adjust their functional composition towards acquisitive strategies in response to drought. For example, long‐term drought experiments in North American grasslands showed that drought tended to induce compositional shifts towards species with acquisitive traits, such as high SLA and LNC (Griffin‐Nolan et al. 2019). Similar findings have also been documented in precipitation reduction experiments conducted in temperate grasslands in southern Germany (Kramp et al. 2022). Evidence from global drylands further suggests that acquisitive strategies can confer survival advantages for plants under drought stress (Gross et al. 2024). These findings collectively imply a shift in community drought response mechanisms from tolerance (conservative growth) to escape and avoidance (acquisitive growth).

In our study, drought conditions fostered the proliferation of annual plants, such as *Corispermum glaucum* and *Salsola abrotanoides*, which increased in relative abundance during drought years (Figure S10) and contributed to increased productivity resistance and temporal stability. These annual plants exhibit traits characteristic of acquisitive species (Funk et al. 2021; Mueller et al. 2024), including high SLA and SRA, low LDMC and elevated LNC and RNC (Figure S15). Data from other semi‐arid grasslands in China further corroborate this pattern, demonstrating that the rise of acquisitive annual species during drought events (Sun et al. 2022). Moreover, global biogeographic studies show that acquisitive annual species are more prevalent in hot and dry regions, suggesting that they are better adapted to these conditions than perennials (Poppenwimer et al. 2023). However, counter evidence also exists. For example, plant communities in North America short grass prairie have been found to shift in composition towards perennial species with more conservative traits (e.g., high LDMC, low SLA) under drought (Wilcox et al. 2021). These discrepancies among studies invite the question to what extent ecological communities respond to drought via alternative strategies, which can be better answered after more data from various ecosystems are available.

Several attributes associated with acquisitive strategies may have contributed to the observed positive relationships between acquisition strategies and productivity resistance/temporal stability. First, plants characterised by acquisitive growth strategies often possess traits associated with drought avoidance, which mitigates water transpiration and reduces drought‐induced mortality and grassland biomass loss. Indeed, acquisitive species in our study grasslands tend to have smaller stomatal area and conductance (see Figure S16), which would reduce water loss. Recent studies also indicate that plants with thinner and more acquisitive leaves tend to close their stomata earlier, leading to more effective prevention of water loss compared to plants with thicker and more conservative leaves (Lubbe et al. 2021). These drought avoidance mechanisms may have mitigated the negative effect of drought on plant survival and growth, contributing to greater resistance and temporal stability. Second, fast‐growing, acquisitive species inherently possess drought escape mechanisms (Franks 2011; Nguyen et al. 2016), which may have also mitigated biomass loss and contributed to increased resistance and temporal stability. Plants employing such strategies exhibit rapid growth, and could complete their life cycles, such as through shorter flowering periods as observed in our study (see Figure S17), before severe drought conditions set in. Finally, seeds of fast‐growing, acquisitive species are often small and possess high dispersal ability, granting them an advantage in habitat colonisation subsequent to their rapid growth and reproduction (Ruppert et al. 2015; Wang et al. 2017). Our analysis shows that seeds of more acquisitive species in our study grasslands are indeed lighter (Figure S17). Their potential higher dispersal ability may have aided in the recovery of drought‐affected grasslands, contributing to increased ecosystem temporal stability.

We found that grassland stability, particularly temporal stability, tended to decline as larger‐sized species became more abundant. This finding supports the idea that larger plants tend to lose more water through transpiration (Farquhar et al. 2002), leading to lower stability under drought stress (Kramp et al. 2022). However, the relationships between size traits and productivity stability in our study were generally weaker than those between economic traits and productivity stability, particularly for temporal stability and resistance, lending support to the perceived importance of economic traits for regulating ecosystem stability (de Bello et al. 2021). This is despite the fact that size traits (e.g., LA and tree height) are often more closely linked to ecosystem functions than economic traits, particularly in large‐scale studies (Li et al. 2020; Guo et al. 2022; Yan, He, et al. 2023). Since size and economic traits represent key dimensions of plant form and function globally (Díaz et al. 2016), an important next step is to investigate how their contributions to ecosystem stability vary across ecosystems.

One important limitation of our study is that the transect plant community data were collected during a single growing season (2018), where different climatic conditions across sampling sites (Figure S4) influenced the species composition of local grassland communities. As such, it is likely that not all drought‐adapting strategies (tolerance, escape and avoidance) were sufficiently abundant at each sampling site to be captured by our ‘snap‐shot’ community survey. This is despite the fact that our transect survey data allowed us to detect significant relationships between plant economics traits and multiple stability dimensions. Future studies should aim to collect plant community and trait data over multiple growing seasons for more robust analyses of the role of different drought‐adapting strategies in regulating ecosystem stability.

Our study presents strong evidence that plant communities dominated by acquisitive growth strategies, as opposed to conservative growth strategies, exhibit greater resistance and temporal stability in semi‐arid grasslands. This novel finding expands our understanding of trait–stability relationships by highlighting the distinct advantages of rapid growing, acquisitive plant species with drought escape and avoidance capabilities, an aspect that has previously been underappreciated. Our study thus illustrates that conservative growth may not be the only strategy that sustains ecosystem stability.

## 5 Author Contributions

L.J. and P.Y. designed the research. P.Y., N.H., G.L., S.C., J.S., and S.W. carried out the field work and collected data. P.Y. and L.J. wrote the manuscript. All authors contributed substantially to the revision of the manuscript.

## Conflicts of Interest

The authors declare no conflicts of interest.

### Peer Review

The peer review history for this article is available at https://www.webofscience.com/api/gateway/wos/peer‐review/10.1111/ele.70110.

## Supporting information


Data S1.

## Data Availability

The data and code associated with this study are available on Figshare and can be accessed via the following DOI: https://doi.org/10.6084/m9.figshare.26391163.v3 (Pu et al. 2025).
