# Supplementary material for: Plant Acquisitive Strategies Promote Resistance and Temporal Stability of Semiarid Grasslands
Source: Ecol Lett. 2025 Apr 3;28(4):e70110. doi: 10.1111/ele.70110 (PMC11967160; doi:10.1111/ele.70110)
Supplement: Supplementary file 1 — Data S1. [file ELE-28-0-s001.docx]

**Supporting Information for**

**Plant acquisitive strategies promote resistance and temporal stability of semiarid grasslands**

Pu Yan ^1^, Nianpeng He ^2^, Marcos Fernández-Martínez ^3^, Xian Yang ^4^, Yiping Zuo ^1^, Hao Zhang ^1^, Jing Wang ^5^, Shiping Chen ^6^, Jian Song ^7^, Guoyong Li ^8^, Enrique Valencia ^9^, Shiqiang Wan ^7^, Lin Jiang ^1*^

^1^ School of Biological Sciences, Georgia Institute of Technology, Atlanta, GA, USA

^2^ Key Laboratory of Sustainable Forest Ecosystem Management–Ministry of Education, Northeast Forestry University, Harbin, China

^3^ CREAF, Cerdanyola del Vallès, Barcelona, Spain

^4^ School of Ecology, Sun Yat-sen University, Guangzhou, China

^5^ Key Laboratory of Ecosystem Network Observation and Modeling, Institute of Geographic Sciences and Natural Resources Research, Chinese Academy of Sciences, Beijing, China

^6^ State Key Laboratory of Vegetation and Environmental Change, Institute of Botany, Chinese Academy of Sciences, Beijing, China

^7^ School of Life Sciences, Institute of Life Science and Green Development, Hebei University, Baoding, China

^8^ International Joint Research Laboratory for Global Change Ecology, School of Life Sciences, Henan University, Kaifeng, China

^9^ Department of Biodiversity, Ecology and Evolution, Faculty of Biological Science, Complutense University of Madrid, Madrid, Spain

*Corresponding author. Lin Jiang (lin.jiang@biology.gatech.edu)

**This file includes:**

1) Text S1 and S2

2) Table S1 – S8

3) Figure S1 – S17

4) Supplementary References

**Supplementary text 1 Measurement of plant traits**

**1) Temporal plant community data (Duolun site)**

**Leaf life span (LLS) and flowering duration:** Five healthy plants of each species were selected at the experimental site. For each plant, two leaves were tagged with small labels to facilitate monitoring. Leaf size was measured daily, and leaf lifespan was estimated as the number of days from the cessation of leaf growth to the point when 80% of the leaves had senesced. Flowering duration was determined by observing the flowering phenology of each plant. The start of flowering was recorded as the day when the first flower fully opened, while the end of flowering was marked as the day when the last flower wilted or set fruit. The duration was calculated as the number of days between these two events, representing the active reproductive phase of the plants. All phenological events, including the start and end of leaf lifespan and flowering duration, were recorded using the Julian day (JD) system, where January 1st is assigned as day 1 of the year. This ensured consistency in temporal data representation and facilitated comparison across years and sites.

**Leaf area (LA):** For each species, we selected 5-10 healthy plants. From each plant, we collected 1-2 mature and healthy leaves for measuring their leaf area (LA) and leaf dry mass (LM). Subsequently, the leaves were immersed in water for 6 hours, and blotted dry with paper towels. The saturated fresh weight of the leaves was then measured using a precision balance. Next, the leaves were scanned for area using a Canon CanoScan LIDE 210 flatbed scanner. Following scanning, the leaves were placed in envelopes and subjected to dehydration in an oven at 105°C for 1 hour, followed by drying to constant weight in a 65°C oven. Finally, the dried leaves were weighed to obtain their dry weight. **Leaf dry matter content (LDMC) and Specific leaf area (SLA)**: **LDMC** was calculated as the ratio of leaf dry weight to leaf saturated fresh weight, with units of g g^-1^. **SLA** was calculated as the ratio of leaf area to leaf dry weight, with units of cm^2^ g^-1^. **Leaf nitrogen concentration (LNC) and root nitrogen concentration (RNC):** Healthy leaves and fine root of each species were collected from five to 30 individual plants and mixed to from a composite sample. After the sample was dried, we used the agate mortar grinder to crush and grind all the dried samples, and the nitrogen concentration was measured using an elemental analyzer (Vario Max C*N* Element Analyser; Elementar). **Plant height (Height):** Unlike other traits, *plant* *height* was directly measured each year during the survey of community composition. For each species, we averaged plant height values across years. **Seed mass:** Mean *seed dry mass* was estimated after collecting seeds from at least five individuals of each species.

**Root depth (RootDepth)** and **root length** (**RootLength**): **RootDepth** was measured using a steel tape measure from the bottom of the hole dug during root excavation to the ground surface, while **RootLength** was obtained by carefully extending the excavated roots to their maximum length and measuring them along a straight line. To ensure accurate measurements, plants were excavated in the field at representative locations within the study site. Healthy, mature individuals of each species were selected, avoiding plants with visible damage or atypical growth patterns to ensure measurements were representative. The soil surrounding the plants was carefully removed using shovels and hand tools, taking care not to damage the roots. Fine roots were separated manually, and roots were rinsed with water to remove residual soil while maintaining their integrity. To ensure the deepest roots were captured, the main root structure was followed to its endpoint, even if it extended beyond the initial hole, with the excavation area expanded as needed, especially for plants with deep taproots or extensive root systems, to avoid truncation. These careful excavation and separation procedures ensured accurate measurements of both root depth and root length, capturing the entire root system as comprehensively as possible.

**Root diameter (RootDiameter)** refers to the diameter of the portion of a plant's root system that comes into contact with the ground (simultaneously serving as the junction between the roots and aboveground stems), measured using electronic calipers, with units in millimeters (mm). Fine roots are defined as roots with a diameter ≤ 2 mm, primarily responsible for nutrient and water uptake. **Fine root diameter** (**FineRootDiameter**) represents the diameter of these roots, measured in millimeters (mm). The **dry matter content of the roots (RDMC)** is calculated as the mass of dried roots divided by the fresh weight of roots at saturation water content (g g^-1^).

**Specific Root Length (SRL) and Specific Root Area (SRA):** Specific root length (SRL) represents the root length per unit mass of fine roots and was calculated by dividing the total length of fine roots by their dry weight, expressed in mm g⁻¹. Specific root area (SRA) was calculated as the ratio of the cross-sectional area of fine roots (calculated as π times the square of the radius) to their dry weight, expressed in mm² g⁻¹. For both traits, fine roots (defined as roots with a diameter less than 2 mm) were carefully separated from the rest of the root system after excavation. The roots were gently washed with water to remove adhering soil particles while minimizing damage. Root measurements were conducted using the WinRHIZO root scanning system (Regent Instruments Inc., Canada), which provided precise measurements of root length and diameter by analyzing scanned root images. This approach avoided the risk of damaging the roots with calipers or other manual methods. For each plant, measurements were conducted on multiple fine roots, and for each species, traits were measured on 5–10 replicate samples. Data were averaged across these replicates to obtain species-level trait values. This procedure ensured the accuracy and representativeness of both SRL and SRA while maintaining the integrity of the fine roots throughout the process.

**2) Grassland transect survey data**

**Height:** The height of plants was directly measured during field surveys of plots. Prior to harvesting, the height of each species within the plots was measured. For each species, 3-5 individuals (exact number depending on the abundance of the species within the plot) were measured for height, and the mean value was calculated to represent the height of that species within the plot.

**LA, LDMC, and SLA:** Six to ten randomly selected healthy leaves of each species were collected and placed on a portable scanner (CanoScan LIDE 110, Japan), where they were flattened with a transparent plastic plate to ensure the leaves were fully stretched. Leaf area (LA, cm²) was determined using ImageJ software (Liu *et al.* 2021). After scanning, the leaves were saturated by immersing them in deionized water for 24 hours at room temperature. Excess water was gently blotted away with tissue paper, and the leaves were immediately weighed to obtain their saturated mass. The leaves were then dried in an oven at 60 °C for 48 hours and reweighed to determine dry mass. Leaf dry matter content (LDMC, g/g) was calculated as the ratio of leaf dry mass to saturated mass. Specific leaf area (SLA, cm²/g) was calculated by dividing leaf area by leaf dry mass. Given the inclusion of LA, SLA, and LDMC, we did not explicitly present leaf dry mass (LM) in the main analyses, as it offers limited additional information.

**RDMC:** the root dry matter content was calculated as the mass of dried roots divided by the fresh weight of roots at saturation water content (g g^-1^). **LNC, RNC, Leaf phosphorus concentration (LPC), and root phosphorus concentration (RPC):** We used the agate mortar grinder to crush and grind all the dried samples, and the leaf nitrogen concentration was measured using an elemental analyzer (Vario Max C*N* Element Analyser; Elementar). The samples that were used for measuring P were acidified with 68% HNO_3_, and then digested using a microwave digestion system. Finally, the P content was determined with an inductively coupled plasma optical emission spectrometer. More detailed information about sampling and measurements of these traits can be found elsewhere (He *et al.* 2019; Liu *et al.* 2021). **Stomatal area and conductance:** For each species, three small pieces of leaf were selected from the pooled sample, with each replicate being photographed twice, capturing both the adaxial and abaxial surfaces, respectively. Finally, the area of the stomata was calculated based on the stomata length and width. Stomatal area was measured using a scanning electron microscope (S–3400N, Hitachi, Japan).

$\text{Stomatal area = stomatal length× stomatal width ×}\frac{\text{π}}{\text{4}}\text{ }\text{ }\text{ }$(1)

Numerous studies (Maxwell *et al.* 2018; Guerrieri *et al.* 2019) have shown that leaf *△^18^O* (*△^18^O_L_*) is affected by leaf water evaporation process, and the variation of stomatal conductance is directly reflected by *△^18^O*. Consequently, akin to prior research (Wang & Wen 2022), we employed *1/△^18^O* as a proxy for stomatal conductance. *△^18^O* is derived from δ^18^O, which is measured using an isotope ratio mass spectrometer coupled with an elemental analyzer (FLASH 2000 HT, Thermo Fisher Scientific, Bremen, Germany) operating in continuous flow mode (253 Plus, Thermo Fisher Scientific, Bremen, Germany).

$\triangle^{18}O_{L}$*=* $\frac{\text{(}\delta^{\text{18}}\text{O}_{\text{L}} \text{-–}{\text{ }\delta}^{\text{18}}\text{O}_{\text{S}}\text{)}}{\text{(1 + }\delta^{\text{18}}\text{O}_{\text{s}}\text{/1000)}}$ (2)

where $\delta^{18}O_{L}$ and $\delta^{18}O_{S}$are the $\delta^{18}O$ of the bulk leaf at the species level and source water, respectively.

**Supplementary text 2 Precipitation during the growing season**

The growing season at the Dulun station spans from May to October, and precipitation data are sourced from an automated meteorological measurement system (HMP45C, Campbell Scientific, Logan, USA) at the study site. For the grassland transect sampling sites spanning nearly 1000 kilometers, high–resolution monthly scale climate data (CRU TS v4.03 database) were acquired from **https://crudata.uea.ac.uk/cru/data/hrg/** with 0.5° spatial resolution for 2000 to 2022. Monthly potential evapotranspiration (PET) data were downloaded from the Global Potential Evapo–Transpiration Climate Database ([**http://www.csi.cgiar.org**](http://www.csi.cgiar.org)).

While the growing season in semi–arid grasslands is typically recognized to span from May to October, the absence of long–term observational data at certain sampling sites prompted us to employ a quantitative approach to ascertain the length of the growing season across grassland transect investigation sites. All consecutive months meeting the following two conditions were determined as months of plant growth: (1) MMT ≥ 5°C and (2) moisture index (MI) ≥ 0.05 (Yan *et al.* 2023). The MI was computed as the ratio of monthly precipitation to PET, serving as an indicator of the monthly water balance across the sampled sites. The length of the growing season quantitatively assessed in this way is in excellent agreement with the length of the growing season observed from plant phenology at the sampling sites with long–term monitoring in semi–arid grasslands. Finally, the growing season precipitation (mm) is calculated as the sum of the accumulated rainfall during the growing season.

**Supplementary Table 1.** Basic geographic and climatic (including annual and growing season [GS]) information, and community △^18^O for sampling sites in Inner Mongolia Plateau (MP).

| Site | Longitude  (°E) | Latitude  (°N) | Elevation  (m) | Temperature  (℃) | | Precipitation  (mm) | | △^18^O (‰) | Dataset |
| --- | --- | --- | --- | --- | --- | --- | --- | --- | --- |
|  |  |  |  | annual | GS | annual | GS |  |  |
| Duolun | 116.28 | 42.03 | 1324 | 2.1 | 12.20 | 378 | 334 | NA | Single site ^†^ |
| MP01 | 123.51 | 44.59 | 144 | 5.10 | 16.60 | 425 | 410 | 32.54±0.90 | Transect sites |
| MP02 | 121.04 | 44.52 | 269 | 5.80 | 16.66 | 393 | 378 | 32.49±0.71 | Transect sites |
| MP03 | 120.33 | 45.11 | 660 | 3.72 | 13.60 | 387 | 372 | 32.28±0.65 | Transect sites |
| MP04 | 118.36 | 44.77 | 1019 | 0.56 | 12.03 | 345 | 320 | 34.23±0.39 | Transect sites |
| MP05 | 116.52 | 44.26 | 1129 | 1.17 | 12.27 | 283 | 267 | 34.22±1.52 | Transect sites |
| MP06 | 116.67 | 43.55 | 1272 | 0.16 | 11.74 | 321 | 304 | 33.00±0.79 | Transect sites |
| MP07 | 117.68 | 44.51 | 1024 | 1.96 | 12.10 | 319 | 298 | 34.31±0.37 | Transect sites |
| MP08 | 114.89 | 44.01 | 1101 | 0.10 | 12.94 | 228 | 219 | 36.17±0.29 | Transect sites |
| MP09 | 113.50 | 43.84 | 1022 | 2.47 | 14.20 | 199 | 190 | 35.59±0.63 | Transect sites |
| MP10 | 112.15 | 43.63 | 955 | 3.69 | 14.87 | 183 | 169 | 35.56±0.21 | Transect sites |

^†^Note that ‘Single site’ refers to temporal plant community data collected from Duolun Station, while ‘Transect sites’ denotes grassland transect investigation sites. △^18^O indicates the enrichment of ^18^O above the source water in leaf organic matter, while its reciprocal (i.e., 1/△^18^O) serves as a proxy for canopy stomatal conductance in grasslands.

**Supplementary Table 2.** The list of plant traits considered in this study

| TraitName | Category | VarName | UnitName | Dataset |
| --- | --- | --- | --- | --- |
| Flowering duration | Phenology | Florescence | days | Single site ^†^ |
| Leaf life span | Phenology | LLS | days | Single site |
| Leaf nitrogen concentration | Chemical | LNC | Percent | Both |
| Leaf phosphorus concentration | Chemical | LPC | Percent | Transect sites |
| Leaf area | Morphological | LA | cm^2^ | Both |
| Leaf dry mass content | Morphological | LDMC | g g^-1^ | Both |
| Plant height | Morphological | Height | cm | Both |
| Specific leaf area | Morphological | SLA | cm^2^ g^-1^ | Both |
| Seed dry mass | Morphological | SeedDryMass | g | Single site |
| Specific root area | Morphological | SRA | cm^2^ g^-1^ | Single site |
| Specific root length | Morphological | SRL | cm g^-1^ | Single site |
| Fine root diameter | Morphological | FineRootDiameter | mm | Single site |
| Fine root length | Morphological | FineRootLength | cm | Single site |
| Root diameter | Morphological | RootDiameter | mm | Single site |
| Root depth | Morphological | RootDepth | cm | Single site |
| Root length | Morphological | RootLength | cm | Single site |
| Root nitrogen concentration | Chemical | RNC | Percent | Both |
| Root phosphorus concentration | Chemical | RPC | mg g^-1^ | Transect sites |
| Root dry mass content | Morphological | RDMC | Percent | Both |
| Stomatal area | Morphological | StomatalArea | μm^2^ | Transect sites |
| Stomatal conductance (1/*△*^18^O) | Metabolic | StomatalConductance | ‰ | Transect sites |

^†^Note that ‘Single site’ refers to temporal plant community data collected from Dunlun Station, while ‘Transect sites’ denotes grassland transect investigation sites.

**Supplementary Table 3.** Rules for using data involving extreme years to calculate resistance and resilience.

| Pre-extreme year | **Extreme year** | Post-extreme year | Resistance | Resilience |
| --- | --- | --- | --- | --- |
| Dry | **Dry** | Dry | Y | N |
| Dry | **Dry** | Normal | Y | Y |
| Dry | **Dry** | Wet | Y | N |
| Normal | **Dry** | Dry | Y | N |
| Normal | **Dry** | Normal | Y | Y |
| Normal | **Dry** | Wet | Y | N |
| Wet | **Dry** | Dry | N | N |
| Wet | **Dry** | Normal | N | N |
| Wet | **Dry** | Wet | N | N |
| Dry | **Wet** | Dry | N | N |
| Dry | **Wet** | Normal | N | N |
| Dry | **Wet** | Wet | N | N |
| Normal | **Wet** | Dry | Y | N |
| Normal | **Wet** | Normal | Y | Y |
| Normal | **Wet** | Wet | Y | N |
| Wet | **Wet** | Dry | Y | N |
| Wet | **Wet** | Normal | Y | Y |
| Wet | **Wet** | Wet | Y | N |

Combinations of three consecutive growing seasons and selection of the growing seasons for calculating resistance and resilience. The method used here follows Chen *et al.* (2023).

**Supplementary Table 4.** Effects (including 95% credible intervals [CI]) of economic and size traits on community stability dimensions (temporal stability [TS], resistance, and resilience) from temporal plant community data.

| **Model** | **Trait** | **Mean** | **CI 97.5** | **CI 2.5** | **p_loo** | **elpd_loo** | **looic** |
| --- | --- | --- | --- | --- | --- | --- | --- |
| **Functional stability** | |  |  |  |  |  |  |
| TS | Economic trait | 0.89 | 1.29 | 0.50 | 2.16 | -7.92 | 15.84 |
| TS | Size trait | -0.79 | -0.29 | -1.31 | 1.97 | -10.08 | 20.17 |
| Resistance | Economic trait | 0.77 | 1.34 | 0.20 | 3.38 | -11.70 | 23.41 |
| Resistance | Size trait | 0.04 | 0.86 | -0.79 | 2.32 | -14.29 | 28.58 |
| Resilience | Economic trait | 0.03 | 0.83 | -0.81 | 2.16 | -14.09 | 28.18 |
| Resilience | Size trait | 0.76 | 1.32 | 0.20 | 1.77 | -10.62 | 21.25 |
| **Compositional stability** | |  |  |  |  |  |  |
| TS | Economic trait | 0.98 | 1.14 | 0.82 | 2.16 | -0.98 | 1.96 |
| TS | Size trait | -0.87 | -0.48 | -1.30 | 3.32 | -9.33 | 18.65 |
| Resistance | Economic trait | 0.88 | 1.31 | 0.46 | 1.90 | -8.43 | 16.86 |
| Resistance | Size trait | -0.83 | -0.35 | -1.33 | 2.23 | -9.51 | 19.02 |
| Resilience | Economic trait | -0.48 | 0.28 | -1.22 | 2.15 | -13.15 | 26.31 |
| Resilience | Size trait | 0.80 | 1.33 | 0.29 | 2.14 | -10.00 | 19.99 |

Economic traits reflect acquisition-conservative strategy tradeoffs, where higher values denote fast strategies and lower values indicate slow strategies. Size traits represent size trade-offs, with higher values indicating greater height and leaf area. Overall ecological stability is represented by the first principal component after dimensionality reduction of multiple stability dimensions by principal component analysis. The term ***elpd_loo*** denotes the Bayesian Leave-One-Out (LOO) estimate of the expected log pointwise predictive density, which is the sum of N individual pointwise log predictive densities. The term ***p_loo*** refers to the difference between elpd_loo and the non-cross-validated log posterior predictive density. The LOO information criterion (***Looic***) is used for model evaluation, with smaller values indicating a better fit.

**Supplementary Table 5.** Effects (including 95% credible intervals [CI]) of individual traits on functional stability dimensions (temporal stability, resistance, and resilience) and comparison of models from temporal plant community data. Results from a subset of optimal models (*△ELPD < 4*).

| **Model** | **Trait** | **Mean** | **CI 97.5** | **CI 2.5** | **looic** | **elpd_diff** | **se_diff** |
| --- | --- | --- | --- | --- | --- | --- | --- |
| TS | LDMC | -0.89 | -1.43 | -0.39 | 15.8 | 0 | 0 |
| TS | Height | -0.79 | -1.46 | -0.14 | 20.2 | -2.2 | 0.5 |
| TS | SRA | 0.79 | 0.09 | 1.48 | 20.9 | -2.6 | 1.6 |
| TS | FineRootLength | -0.75 | -1.52 | -0.03 | 22 | -3.1 | 1.2 |
| TS | SRL | 0.70 | -0.08 | 1.48 | 23.2 | -3.7 | 1.4 |
| Resistance | RNC | 0.77 | 0.08 | 1.49 | 23.4 | 0 | 0 |
| Resistance | RootDiameter | 0.67 | -0.13 | 1.38 | 23.5 | 0 | 3.9 |
| Resistance | LNC | 0.50 | -0.41 | 1.39 | 26.5 | -1.5 | 2.2 |
| Resistance | LDMC | -0.22 | -1.23 | 0.75 | 27.6 | -2.1 | 2.5 |
| Resilience | RootDiameter | -0.39 | -1.34 | 0.55 | 26.5 | 0 | 0 |
| Resilience | LA | 0.49 | -0.45 | 1.45 | 26.6 | -0.1 | 1.6 |
| Resilience | RootDepth | 0.30 | -0.72 | 1.27 | 27 | -0.2 | 1.1 |
| Resilience | Height | -0.19 | -1.25 | 0.84 | 27.6 | -0.5 | 1.1 |
| Resilience | LDMC | -0.14 | -1.18 | 0.88 | 28.2 | -0.8 | 1.2 |

The LOO (Leave-One-Out) information criterion (***Looic***) is used for model evaluation, with smaller values indicating a better fit. ***eldp_diff*** is the difference in eldp between a pair of models. ***se_diff*** is the standard error of component-wide differences of eldp between a pair of models.

**Supplementary Table 6.** Effects (including 95% credible intervals [CI]) of individual traits on community composition stability dimensions (temporal stability [TS], resistance, and resilience) and comparison of models from temporal plant community data. Results from a subset of optimal models (*△ELPD < 4*).

| **Model** | **Trait** | **Mean** | **CI 97.5** | **CI 2.5** | **looic** | **elpd_diff** | **se_diff** |
| --- | --- | --- | --- | --- | --- | --- | --- |
| TS | LLS | -0.059 | -0.035 | -0.082 | -30.33 | 0 | 0 |
| TS | SRA | 0.059 | 0.083 | 0.036 | -28.48 | -0.9 | 1.5 |
| TS | RDMC | -0.05 | -0.031 | -0.084 | -27.84 | -1.2 | 0.8 |
| TS | SLA | 0.059 | 0.082 | 0.036 | -27.53 | -1.4 | 2.6 |
| TS | RootDepth | -0.055 | -0.024 | -0.083 | -25.08 | -2.6 | 1.6 |
| TS | RootLength | -0.054 | -0.022 | -0.084 | -23.84 | -3.2 | 2.1 |
| Resistance | LLS | -0.048 | -0.019 | -0.077 | -26.85 | 0 | 0 |
| Resistance | SRL | 0.047 | 0.076 | 0.017 | -26.07 | -0.4 | 1.8 |
| Resistance | SRA | 0.047 | 0.081 | 0.018 | -25.72 | -0.6 | 1.7 |
| Resistance | RootDepth | -0.045 | -0.012 | -0.079 | -25.16 | -0.8 | 1.0 |
| Resistance | RDMC | -0.045 | -0.013 | -0.078 | -25.00 | -0.9 | 0.5 |
| Resistance | SLA | 0.046 | 0.077 | 0.014 | -24.66 | -1.1 | 0.8 |
| Resistance | RootLength | -0.044 | -0.011 | -0.078 | -24.38 | -1.2 | 1.3 |
| Resistance | SeedDryMass | -0.037 | 0.001 | -0.078 | -20.20 | -3.3 | 1.3 |
| Resilience | RootDiameter | -0.015 | 0.006 | -0.035 | -32.07 | 0 | 0 |
| Resilience | SRA | -0.017 | 0.002 | -0.036 | -33.36 | -2.1 | 1.2 |
| Resilience | SRL | 0.008 | 0.033 | -0.015 | -29.70 | -2.7 | 1.4 |
| Resilience | SLA | -0.012 | 0.009 | -0.032 | -31.09 | -3.2 | 1.3 |
| Resilience | RootLength | 0.010 | 0.031 | -0.012 | -30.73 | -3.4 | 1.7 |
| Resilience | RootDepth | 0.022 | 0.036 | 0.007 | -37.47 | -3.9 | 1.7 |

The LOO (Leave-One-Out) information criterion (***Looic***) is used for model evaluation, with smaller values indicating a better fit. ***eldp_diff*** is the difference in eldp between a pair of models. ***se_diff*** is the standard error of component-wide differences of eldp between a pair of models.

**Supplementary Table 7.** Effects (including 95% credible intervals [CI]) of economic and size traits on temporal stability [TS], resistance, and resilience of productivity from grassland transect survey data.

| **Model** | **Trait** | **Mean** | **CI 97.5** | **CI 2.5** | **p_loo** | **elpd_loo** | **looic** |
| --- | --- | --- | --- | --- | --- | --- | --- |
| TS | Economic trait | 0.61 | 0.75 | 0.46 | 2.94 | -97.35 | 194.70 |
| TS | Size trait | -0.03 | 0.15 | -0.21 | 2.80 | -115.88 | 231.77 |
| Resistance | Economic trait | 0.46 | 0.63 | 0.30 | 2.46 | -106.08 | 212.15 |
| Resistance | Size trait | -0.34 | -0.16 | -0.52 | 2.34 | -111.32 | 222.26 |
| Resilience | Economic trait | 0.68 | 0.82 | 0.54 | 2.62 | -90.79 | 181.58 |
| Resilience | Size trait | -0.02 | 0.17 | -0.20 | 2.32 | -115.75 | 231.49 |

Economic traits reflect acquisition-conservative strategy tradeoffs, where higher values denote fast strategies and lower values indicate slow strategies. Size traits represent size trade-offs, with higher values indicating greater height and leaf area. The term ***elpd_loo*** denotes the Bayesian Leave-One-Out (LOO) estimate of the expected log pointwise predictive density, which is the sum of N individual pointwise log predictive densities. The term ***p_loo*** refers to the difference between elpd_loo and the non-cross-validated log posterior predictive density. The LOO information criterion (***Looic***) is used for model evaluation, with smaller values indicating a better fit.

**Supplementary Table 8.** Effects (including 95% credible intervals [CI]) of individual traits on temporal stability (TS), resistance, and resilience of productivity from grassland transect survey data.

| **Model** | **Trait** | **Mean** | **CI 97.5** | **CI 2.5** | **p_loo** | **elpd_loo** | **looic** |
| --- | --- | --- | --- | --- | --- | --- | --- |
| TS | Height | -0.08 | 0.10 | -0.27 | 2.57 | -115.52 | 231.05 |
| TS | LA | 0.02 | 0.20 | -0.16 | 2.79 | -115.95 | 231.89 |
| TS | SLA | 0.66 | 0.80 | 0.52 | 2.49 | -92.65 | 185.31 |
| TS | LDMC | -0.74 | -0.61 | -0.87 | 2.35 | -83.91 | 167.83 |
| TS | RDMC | -0.03 | 0.16 | -0.22 | 3.07 | -116.08 | 232.17 |
| TS | LNC | 0.33 | 0.51 | 0.15 | 2.71 | -111.34 | 222.68 |
| TS | LPC | 0.38 | 0.56 | 0.20 | 3.13 | -109.84 | 219.68 |
| TS | RNC | 0.44 | 0.62 | 0.27 | 2.51 | -107.08 | 214.16 |
| TS | RPC | 0.21 | 0.39 | 0.02 | 2.58 | -114.06 | 228.12 |
| Resistance | Height | -0.019 | -0.010 | -0.028 | 2.35 | 126.53 | -253.06 |
| Resistance | LA | -0.015 | -0.005 | -0.024 | 2.33 | 124.23 | -248.47 |
| Resistance | SLA | 0.022 | 0.031 | 0.014 | 2.30 | 129.16 | -258.32 |
| Resistance | LDMC | -0.032 | -0.024 | -0.040 | 2.42 | 139.23 | -278.46 |
| Resistance | RDMC | -0.010 | 0.000 | -0.020 | 2.42 | 122.38 | -244.77 |
| Resistance | LNC | 0.016 | 0.026 | 0.007 | 2.30 | 125.19 | -250.38 |
| Resistance | LPC | 0.013 | 0.023 | 0.004 | 2.38 | 123.61 | -247.22 |
| Resistance | RNC | 0.011 | 0.021 | 0.002 | 2.28 | 122.76 | -245.51 |
| Resistance | RPC | 0.015 | 0.024 | 0.006 | 2.32 | 124.21 | -248.42 |
| Resilience | Height | -0.005 | 0.012 | -0.022 | 2.30 | 79.66 | -159.32 |
| Resilience | LA | 0.001 | 0.017 | -0.015 | 2.07 | 79.74 | -159.48 |
| Resilience | SLA | 0.058 | 0.070 | 0.045 | 2.66 | 102.89 | -205.79 |
| Resilience | LDMC | -0.049 | -0.036 | -0.063 | 2.66 | 94.34 | -188.68 |
| Resilience | RDMC | -0.042 | -0.028 | -0.057 | 1.78 | 90.53 | -181.07 |
| Resilience | LNC | 0.041 | 0.055 | 0.027 | 2.58 | 89.55 | -179.10 |
| Resilience | LPC | 0.068 | 0.078 | 0.058 | 2.36 | 116.70 | -233.39 |
| Resilience | RNC | 0.034 | 0.049 | 0.019 | 2.67 | 86.06 | -172.13 |
| Resilience | RPC | 0.045 | 0.059 | 0.031 | 2.13 | 92.11 | -184.22 |

The term ***elpd_loo*** denotes the Bayesian Leave-One-Out (LOO) estimate of the expected log pointwise predictive density, which is the sum of N individual pointwise log predictive densities. The term ***p_loo*** refers to the difference between elpd_loo and the non-cross-validated log posterior predictive density. The LOO information criterion (***Looic***) is used for model evaluation, with smaller values indicating a better fit.


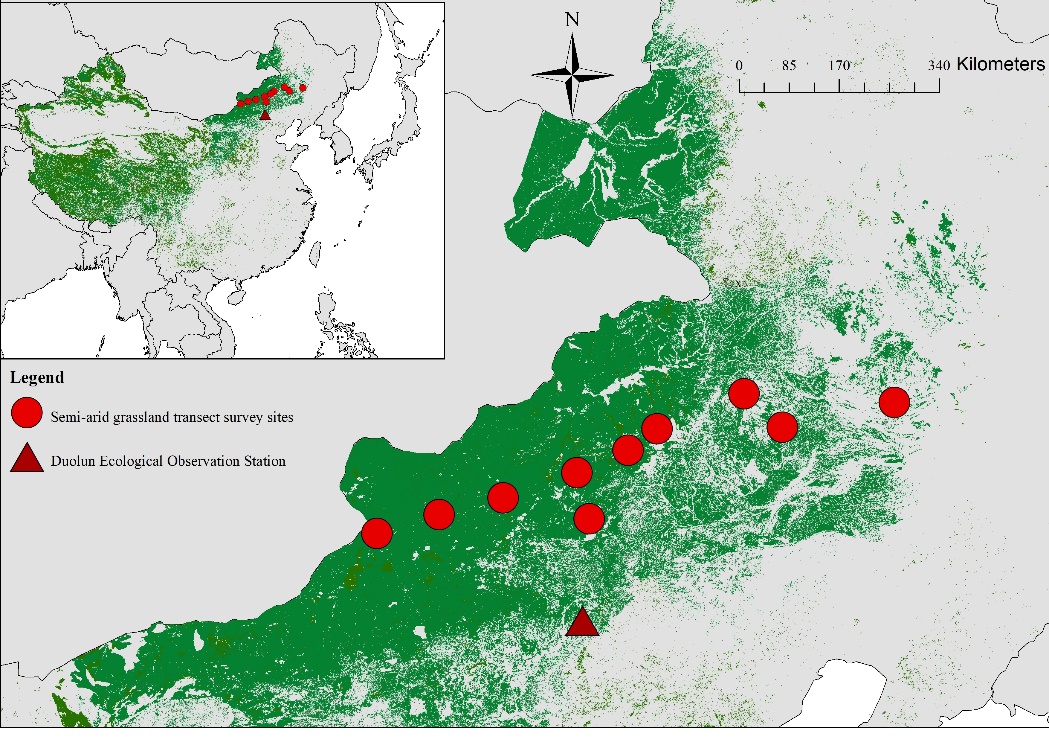


**Figure S1. Distribution map of sampling sites in this study.** Green pixels represent areas with grassland vegetation in China. Circles denote grassland transect survey sites, while the triangle indicates long-term monitoring sites established at the Duolun Ecological Station. As illustrated in the figure, all sites are located within the semi-arid grassland region of Inner Mongolia, China.


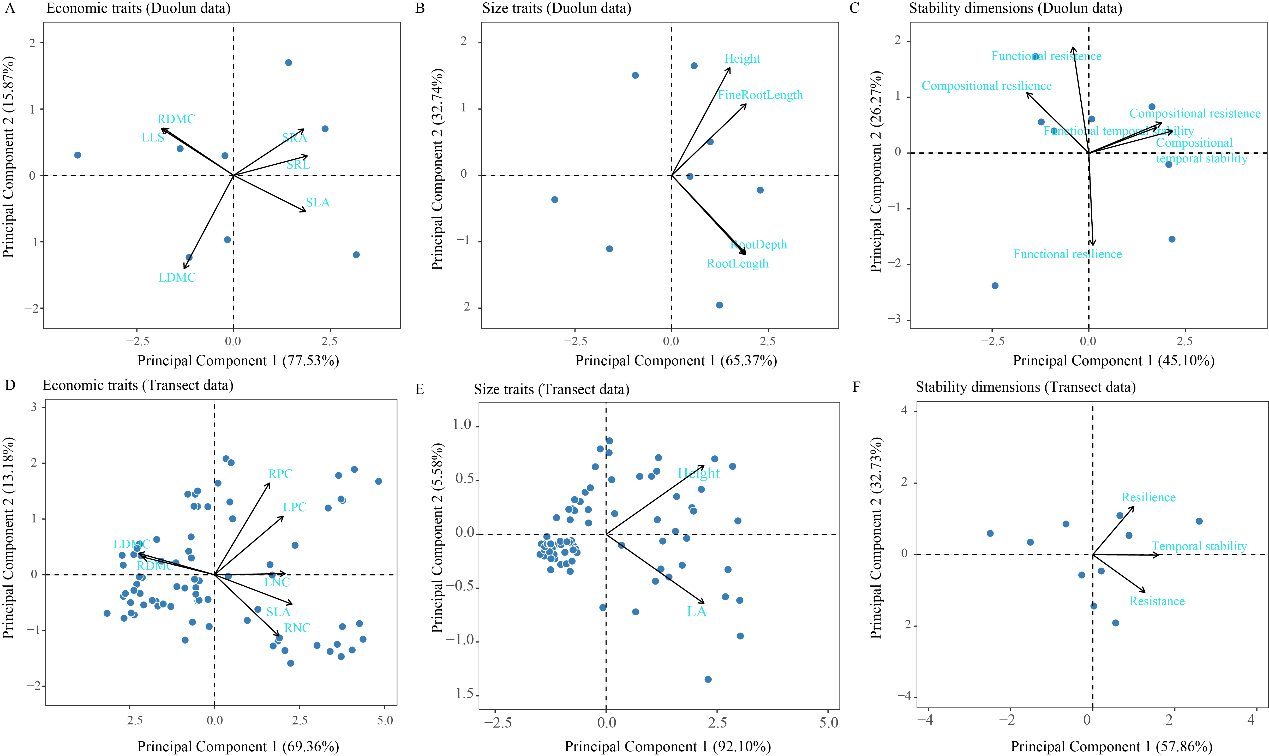


**Figure S2.** **Results of** **principal component analysis (PCA) of economic traits (A, D), size traits (B, E), and stability dimensions (C, F).** Panels A to C represent PCA of data from the single site at Duolun Ecological Station, while panels D to F represent PCA of data from multiple sites along the grassland transect.


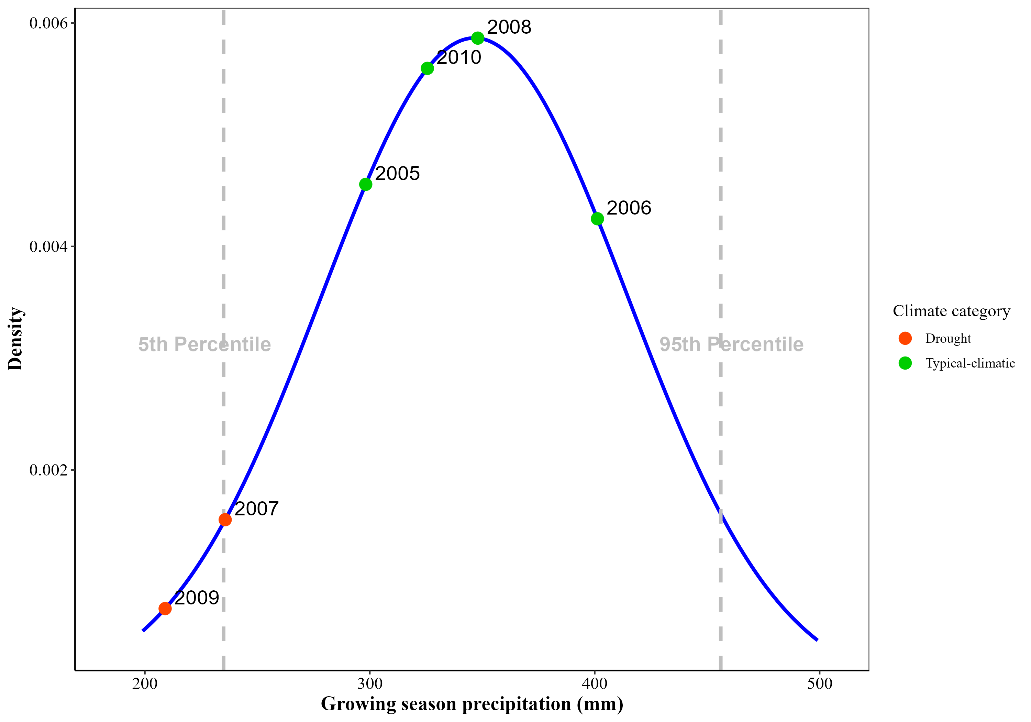


**Figure S3. The frequency distribution of growing season precipitation (May–October) at Duolun Ecological Station, based on meteorological observations recorded from 1953 to 2010.** The fitted curve represents the precipitation distribution derived from these long-term records. The data indicate that 2007 and 2009 were drought years, and the remaining study years were typical-climatic years.


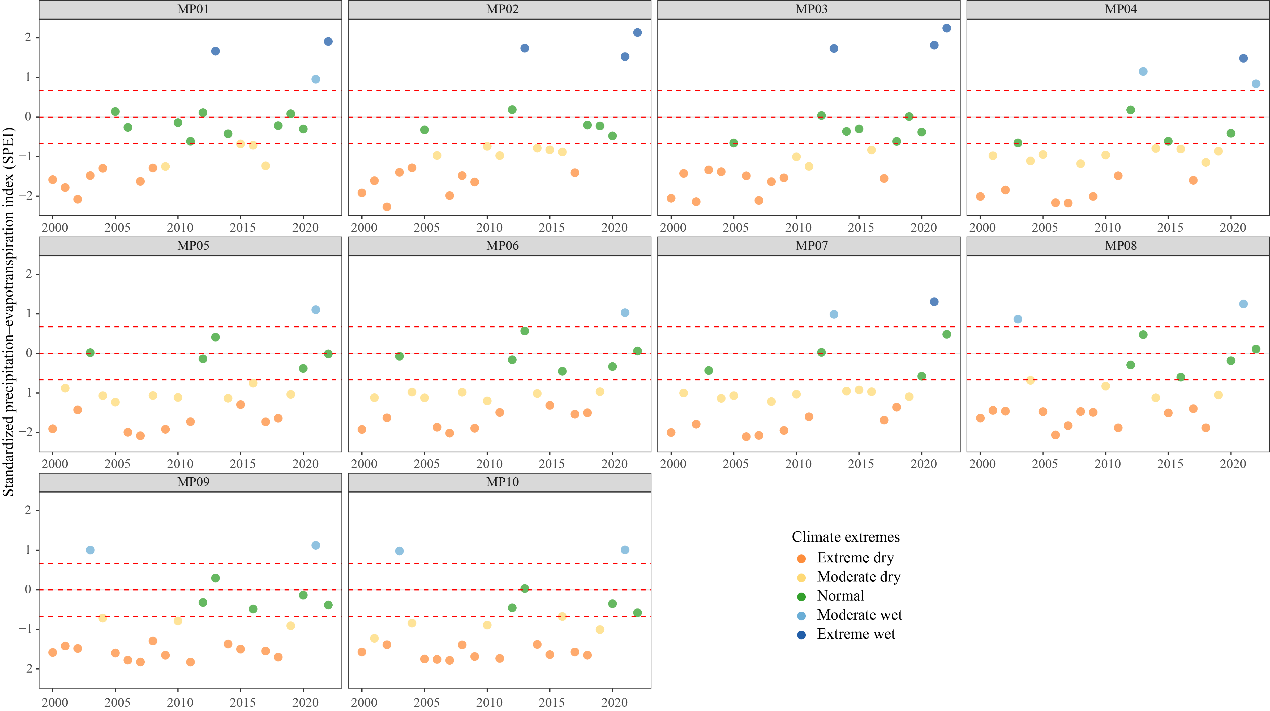


**Figure S4. Climate classification of each site within the Inner Mongolia Plateau (MP) grassland transect from 2000 to 2022, based on the Standardized Precipitation-Evapotranspiration Index (SPEI).** The upper and lower red dashed lines represent thresholds of 0.67 and – 0.67, respectively. Values greater than 0.67 indicate a wet year (occurring approximately once every four years), while values greater than 1.28 indicate an extremely wet year (occurring approximately once per decade). Conversely, values less than – 0.67 indicate a dry year (occurring approximately once every four years), and values less than – 1.28 indicate an extremely dry year (occurring approximately once per decade). The circles in the figure are color-coded to correspond to the five distinct climate scenarios.


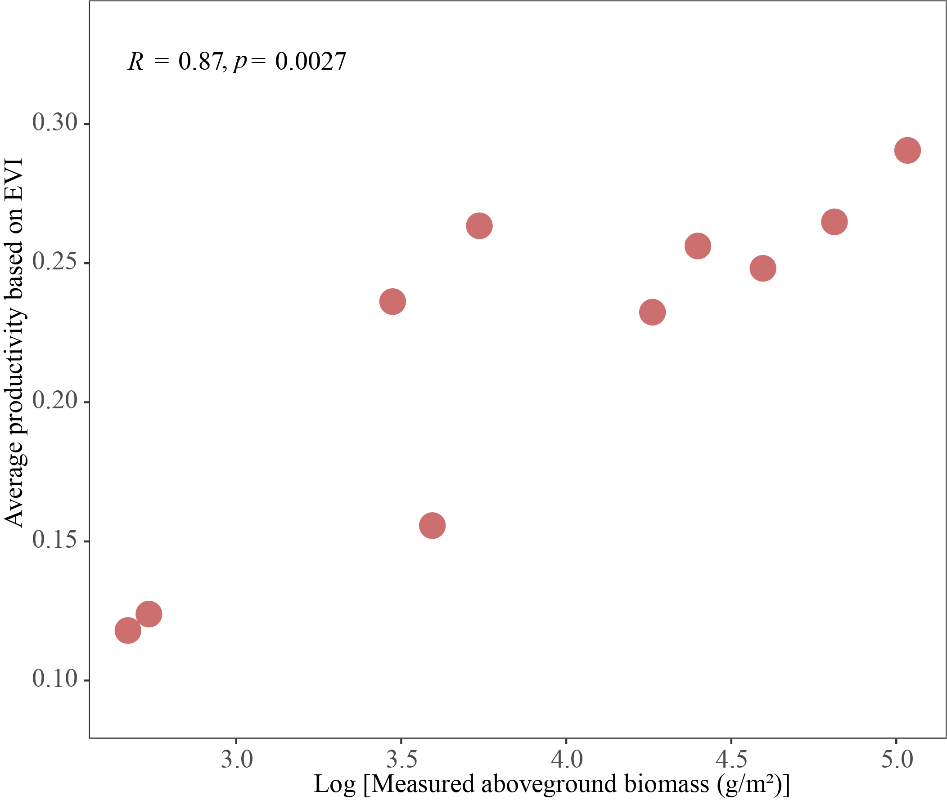


**Figure S5. The relationship between aboveground biomass, as determined through field measurements, and EVI data derived from the MOD13Q1 product.** The correlation coefficient and its *P*-value were calculated using Spearman's non-parametric statistics.


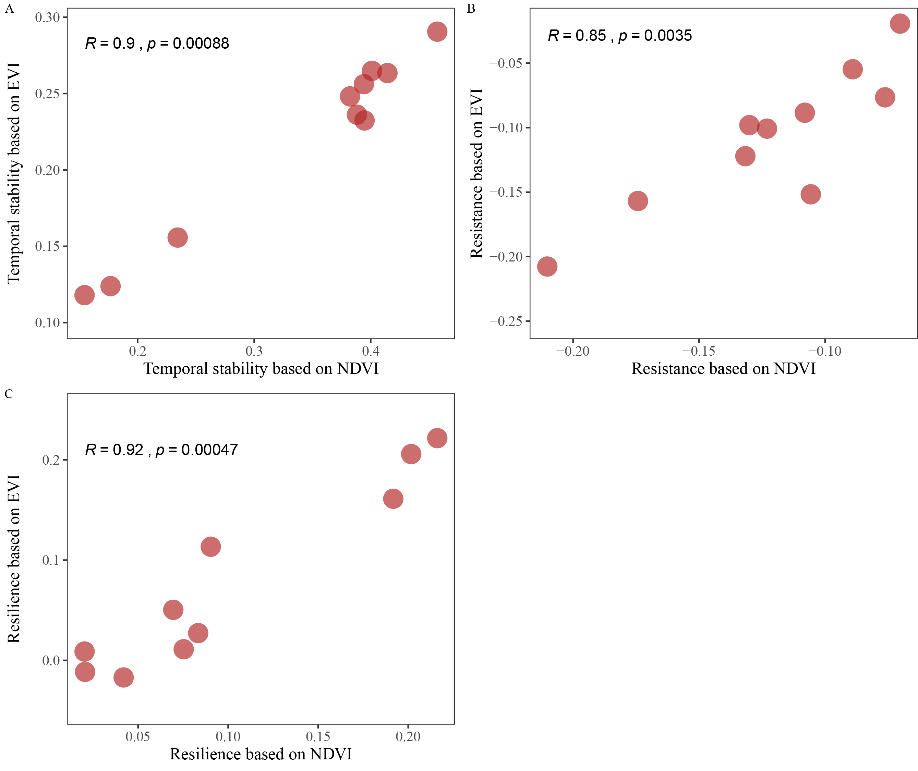


**Figure S6. The relationships between stability dimensions calculated based on EVI and NDVI.** The correlation coefficient and its *P*-value are derived from Spearman non-parametric statistics.


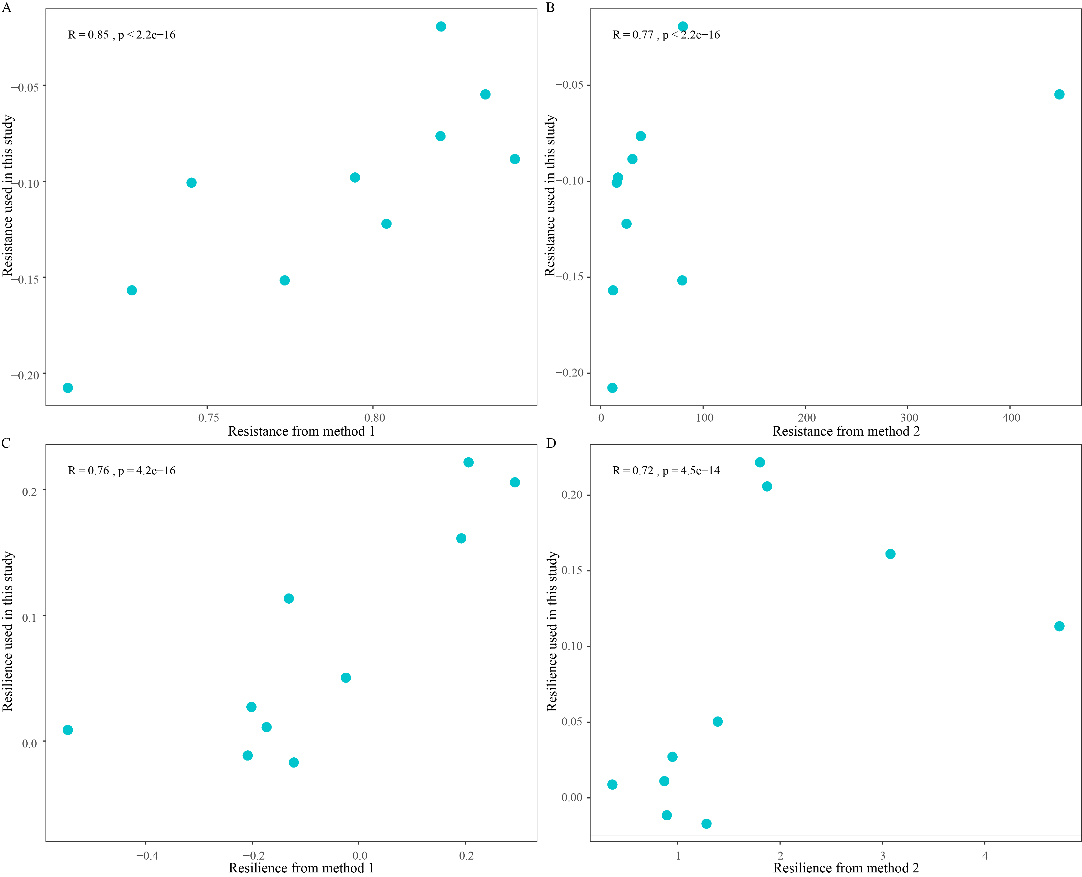


**Figure S7. Relationships between stability (resistance and resilience) metrics derived from different methodologies.** The correlation coefficient and its *P*-value are derived from Spearman non-parametric statistics. The first method follows Liu *et al.* (2022), with the formulas: $\text{Resistance}\text{ }\text{=}\text{ }1-\frac{2 \times|{\overline{\mathrm{Ecosystem}}}_{\mathrm{normal}}- \mathrm{Ecosystem}_{\mathrm{extreme}}|}{\mathrm{Ecosystem}_{\mathrm{normal}} + |\mathrm{Ecosystem}_{\mathrm{normal}} - \mathrm{Ecosystem}_{\mathrm{extreme}}|}$

$$\text{Resilience}\text{ }\text{=}\text{ }\frac{2 \times|{\overline{\mathrm{Ecosystem}}}_{\mathrm{normal}}- \mathrm{Ecosystem}_{\mathrm{extreme}}|}{\left( |{\overline{\mathrm{Ecosystem}}}_{\mathrm{normal}} - \mathrm{Ecosystem}_{\mathrm{extreme}}| + |{\overline{\mathrm{Ecosystem}}}_{\mathrm{normal}} - \mathrm{Ecosystem}_{post-extreme}| \right)}-1$$

The second method adheres to Isbell *et al.* (2015), with the formulas: $\text{Resistance}\text{ }\text{=}\text{ }\frac{{\overline{\mathrm{Ecosystem}}}_{\mathrm{normal}}}{|\mathrm{Ecosystem}_{\mathrm{extreme}} - {\overline{\mathrm{Ecosystem}}}_{\mathrm{normal}}|}$

$$\text{Resilience}\text{ }\text{=}\text{ }\frac{\left| \text{Ecosystem}_{\mathrm{extreme}}- {\overline{\mathrm{Ecosystem}}}_{\mathrm{normal}} \right|}{\left| \text{Ecosystem}_{post-extreme} - {\overline{\mathrm{Ecosystem}}}_{\mathrm{normal}} \right|}$$

${\overline{\mathrm{Ecosystem}}}_{\mathrm{normal}}$ is ecosystem productivity averaged over all normal growing seasons, $\mathrm{Ecosystem}_{\mathrm{extreme}}$represents ecosystem productivity in drought years and $\text{Ecosystem}_{post-extreme}$ represents ecosystem productivity in the year following the drought.


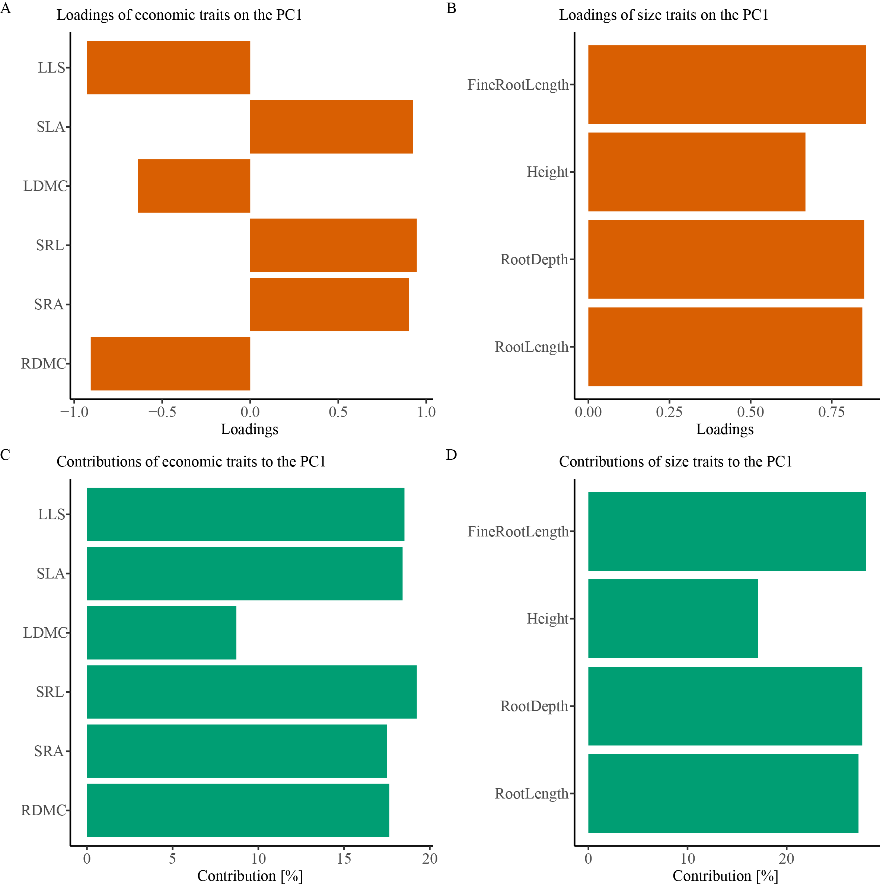


**Figure S8. Loadings of the first principal component from the principal component analysis (PCA) of plant economic (A) and size (B) traits in the temporal community dataset, along with the relative contribution of each economic (C) and size (D) trait.**


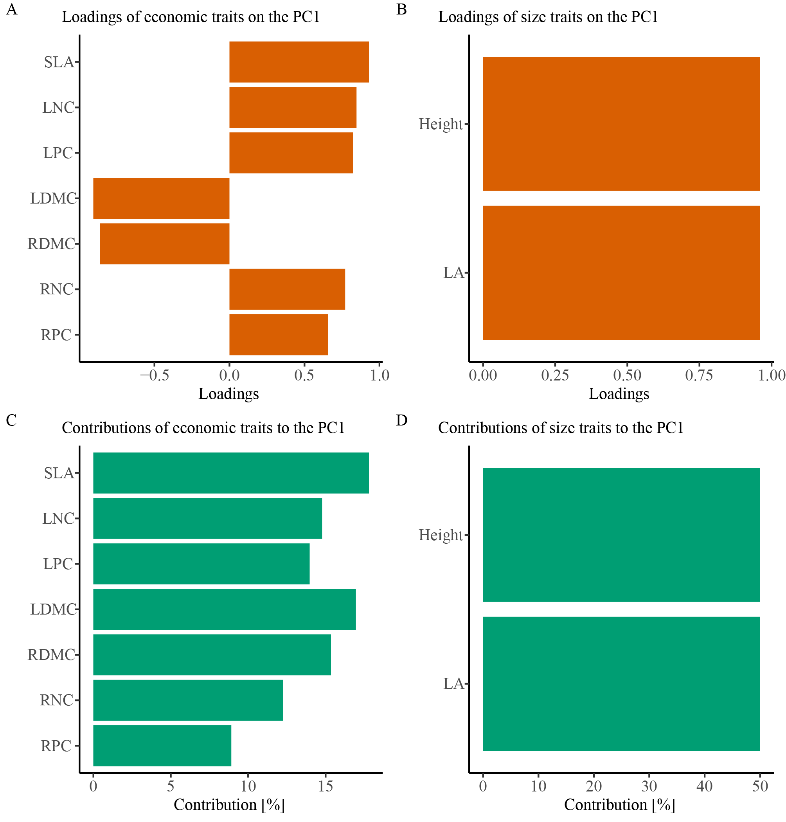


**Figure S9. Loadings of the first principal component from the principal component analysis (PCA) of plant economic (A) and size (B) traits in the transect survey dataset, along with the relative contribution of each economic (C) and size (D) trait.**


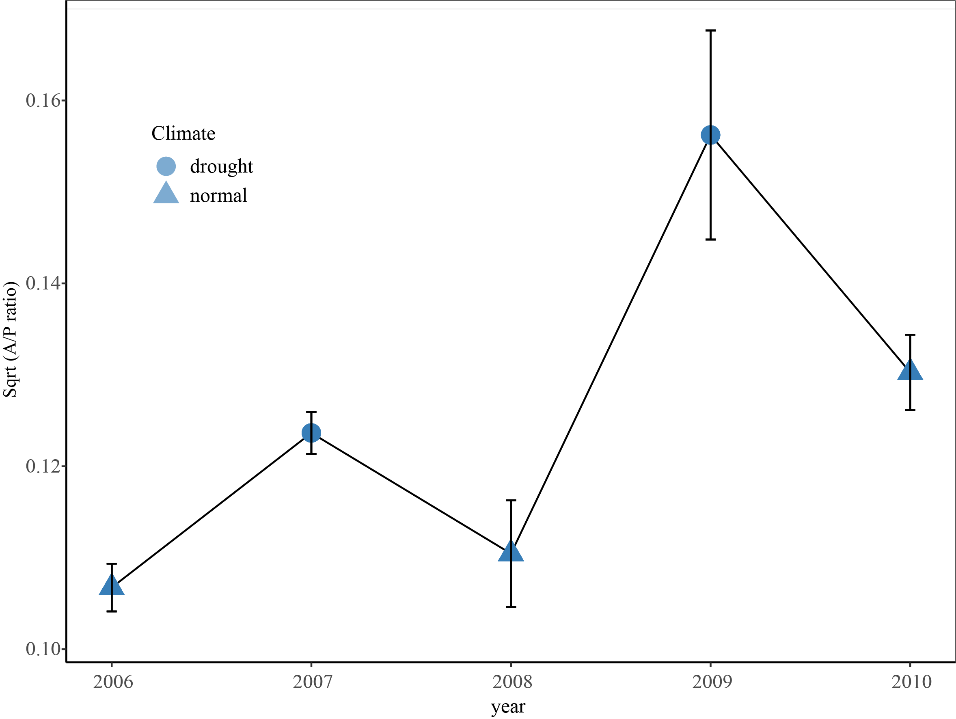


**Figure S10. Temporal trends in the annual-to-perennial plant ratio (A/P ratio) at Duolun Ecological Station**. Error bars denote the standard error.


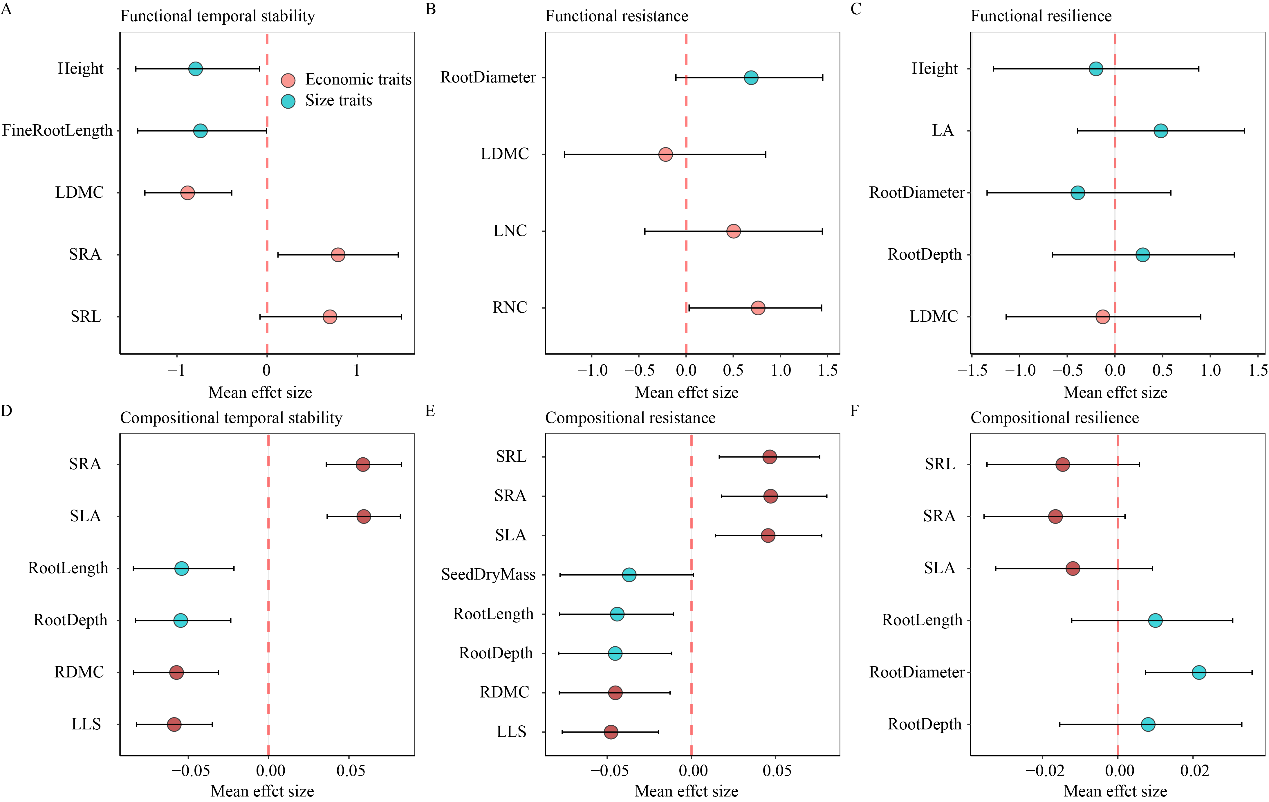


**Figure S11. The effects of individual traits on functional (A–C) and compositional (D–F) temporal stability, resistance, and resilience from temporal plant community data.** Circles represent mean standardized effect sizes with 95% credible intervals (error bars), derived from bivariate Bayesian models. The traits displayed are from the best models (*△ELPD < 4*, Table S4). FineRootLength, fine root length; Height, plant height; LA, leaf area; LDMC, leaf dry mass content; LLS, leaf life span; LNC, leaf nitrogen concentration; RDMC, root dry mass content; RNC, root nitrogen concentration; RootDepth, root depth; RootDiameter, root diameter; RootLength, root length; SLA, specific leaf area; SRA, specific root area; SRL, specific root length.


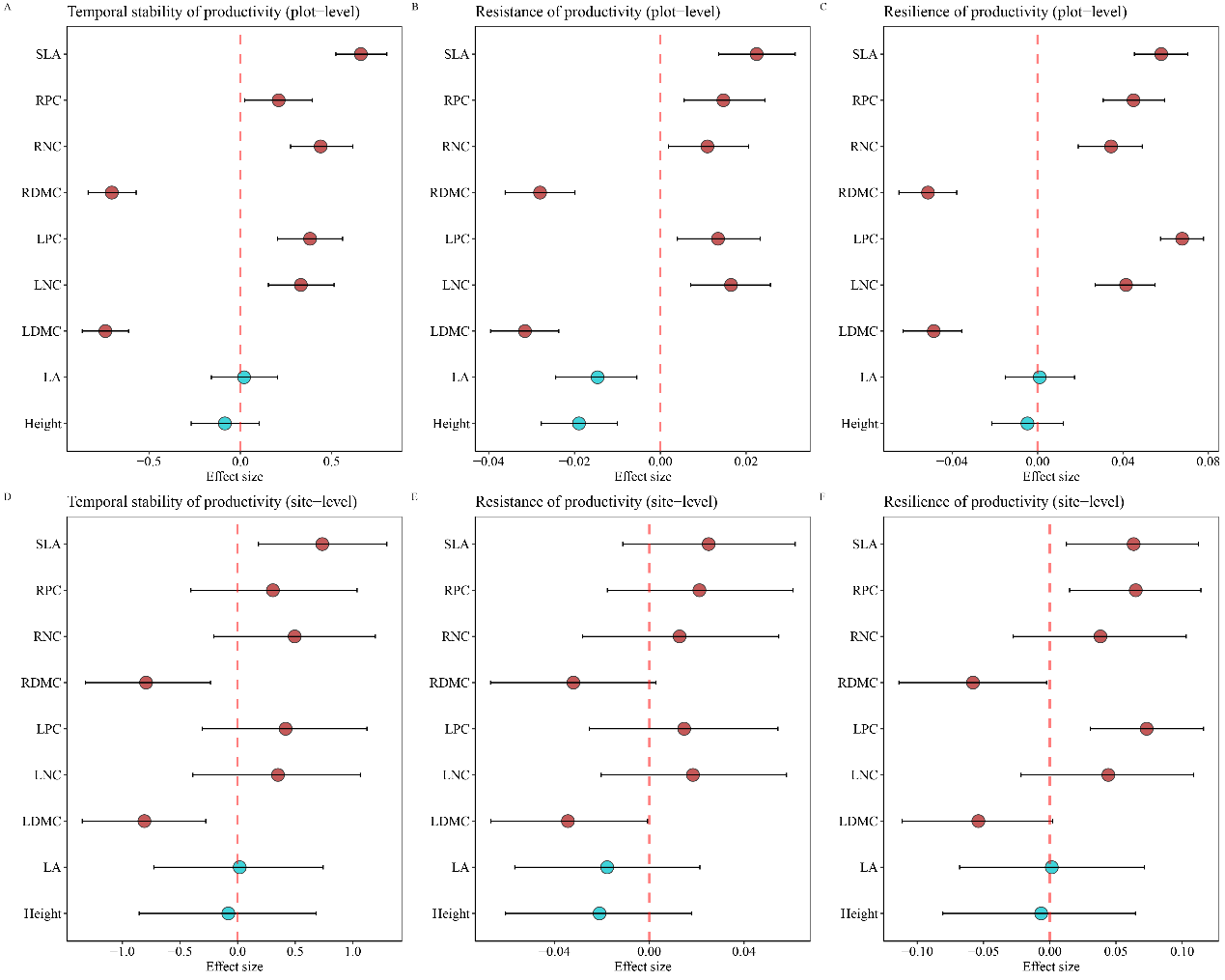


**Figure S12 The effects of individual traits on functional (i.e., productivity) temporal stability (A), resistance (B), and resilience (C), based on grassland transect survey data.** Panels A–C present plot-level analyses (n = 80), while Panels D–F present site-level analyses (n = 10). Circles represent mean standardized effect sizes with 95% credible intervals (error bars), derived from Bayesian models. Individual traits include SLA (specific leaf area), RPC (root phosphorus concentration), RNC (root nitrogen concentration), RDMC (root dry mass content), LPC (leaf phosphorus concentration), LNC (leaf nitrogen concentration), LDMC (leaf dry mass content), LA (leaf area), and Height (plant height).


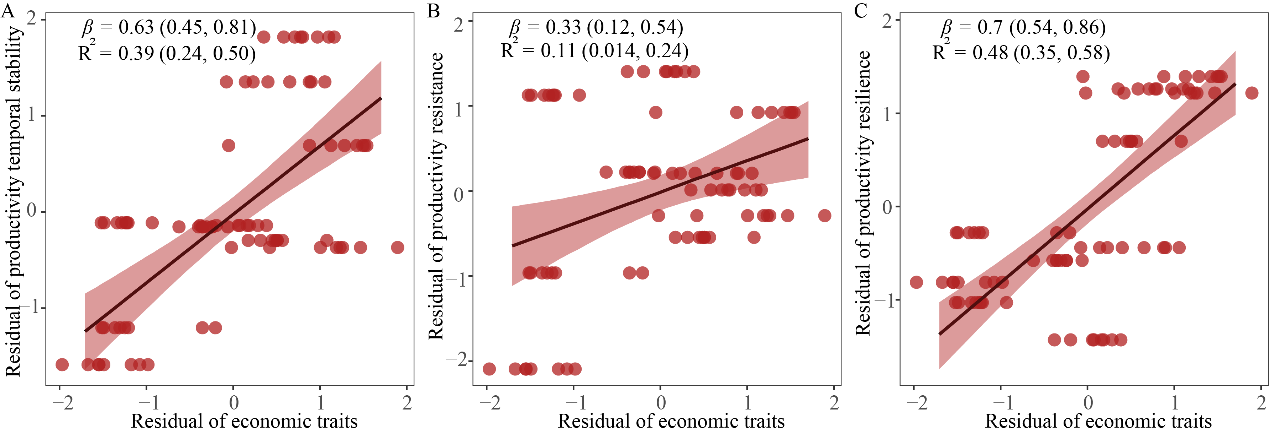


**Figure S13. The relationships between temporal stability, resistance, and resilience of productivity and economic traits while controlling for the influence of growing season precipitation.** The regression lines, derived from Bayesian bivariate regression model, illustrate the predicted relationships between economic traits and each stability dimension, adjusted for precipitation effects. Shaded areas around the regression lines represent 95% credible intervals (CIs), providing a visual measure of uncertainty. The regression coefficients (***β***), extracted from the posterior distributions of the Bayesian model, quantify the strength and direction of these relationships. Economic traits reflect acquisition–conservative strategy trade-offs, where higher values denote acquisitive strategies and lower values conservative strategies.


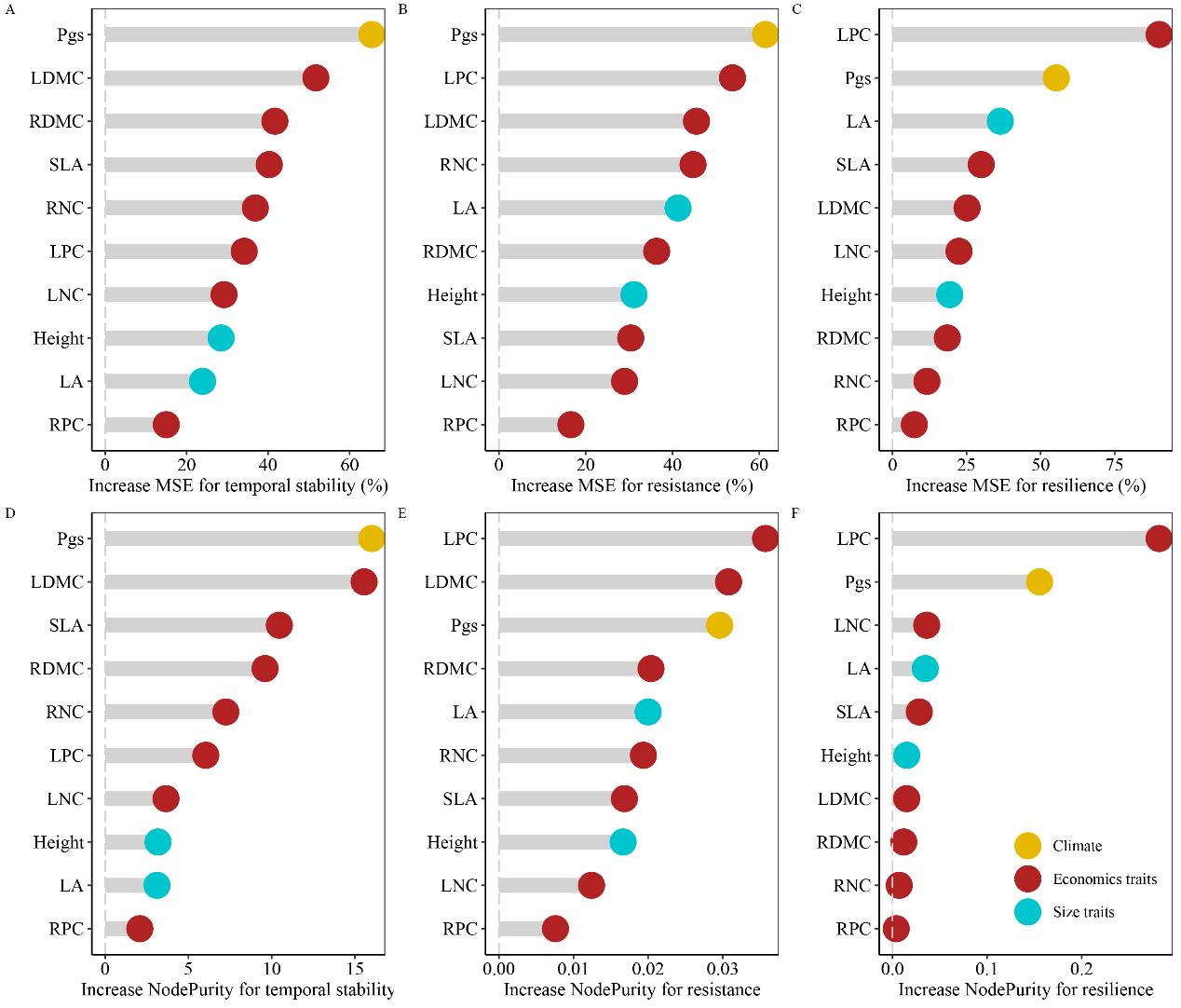


**Figure S14. Relative importance of growing season precipitation and individual traits on temporal stability (panels A and D), resistance (B and E), and resilience (C and F) of different explanatory variables calculated using random forest models based on grassland transect survey data.** Panels A-C represent the use of the ***Permutation Importance (OOB Data)*** method, which assesses variable importance by measuring the increase in prediction error when a variable's values are permuted. Panels D-F represent the use of the ***Decrease in Node Impurity*** method, which evaluates importance by calculating the average reduction in node impurity (Gini index for classification, and residual sum of squares for regression) from splits involving the variable. Pgs, growing season precipitation; SLA, specific leaf area; RPC, root phosphorus concentration; RNC, root nitrogen concentration; RDMC, root dry mass content; LPC, leaf phosphorus concentration; LNC, leaf nitrogen concentration; LDMC, leaf dry mass content; LA, leaf area; Height, plant height.


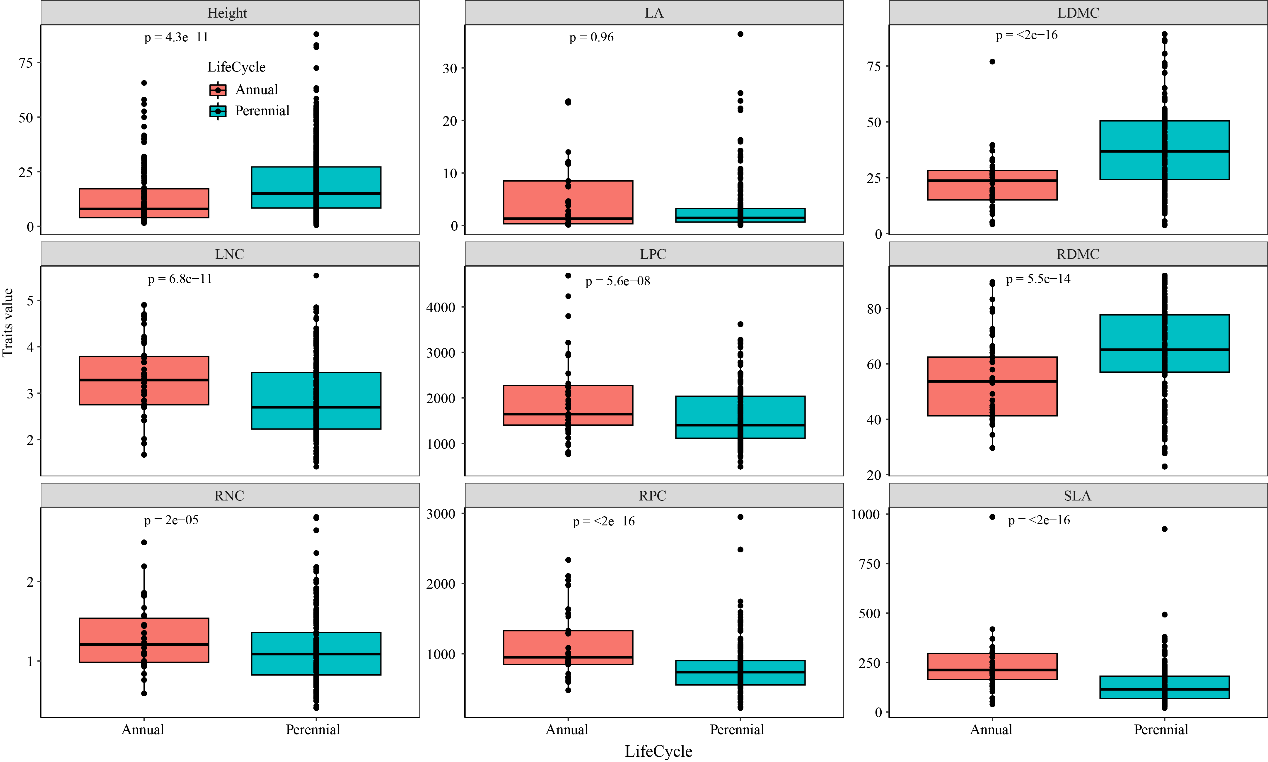
**Figure S15. Comparison of mean trait values between annual and perennial plants.** The boxplot illustrates the distribution of trait values, where the boxes represent the interquartile range (IQR) encompassing the middle 50% of the data, the horizontal line within each box indicates the median value, and the whiskers extend to the smallest and largest values within 1.5 times the IQR. *P* values reflect the statistical significance of differences in mean trait values between annual and perennial plants, as determined by the Wilcoxon Rank Sum Test, with smaller *P* values (e.g., < 0.05) indicating significant differences in trait distributions.


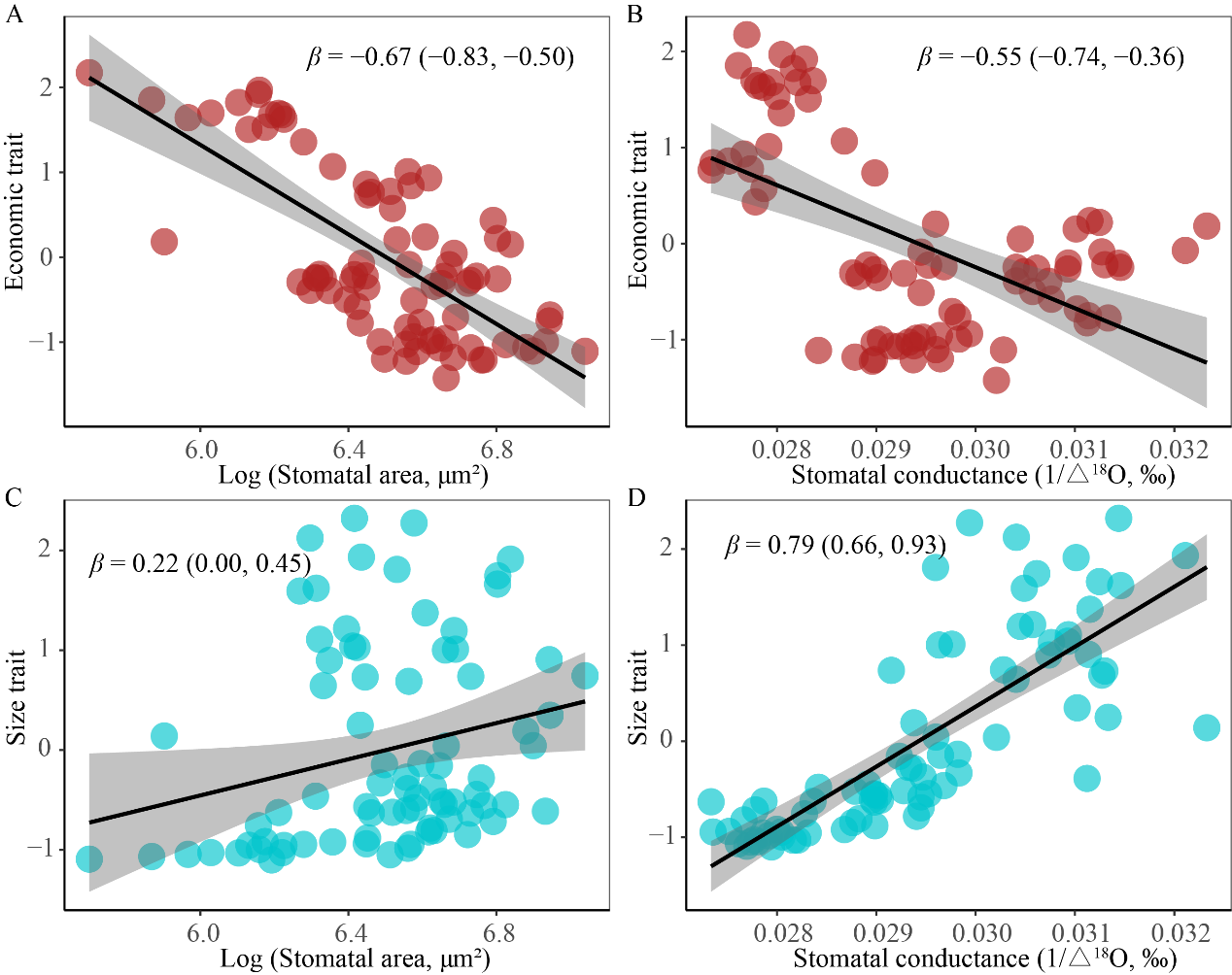


**Figure S16. The relationship between economic and size traits and stomatal area and conductance (1/Δ^18^O) based on the grassland transect survey data.** Economic traits reflect acquisition–conservative strategy trade-offs, where higher values denote fast strategies emphasizing rapid resource acquisition and lower values indicate slow strategies prioritizing resource conservation. Size traits represent size trade-offs, with higher values indicating greater plant height and larger leaf area. Δ^18^O indicates the enrichment of ^18^O above the source water in leaf organic matter, while its reciprocal (1/Δ^18^O) serves as a proxy for canopy stomatal conductance in grasslands. The regression lines illustrate the predicted relationships between traits and stomatal area or conductance, derived from a bivariate Bayesian regression model. The beta coefficients (***β***) represent the posterior means of the regression coefficients, quantifying the magnitude and direction of these relationships, with positive values indicating a direct association and negative values suggesting an inverse relationship.


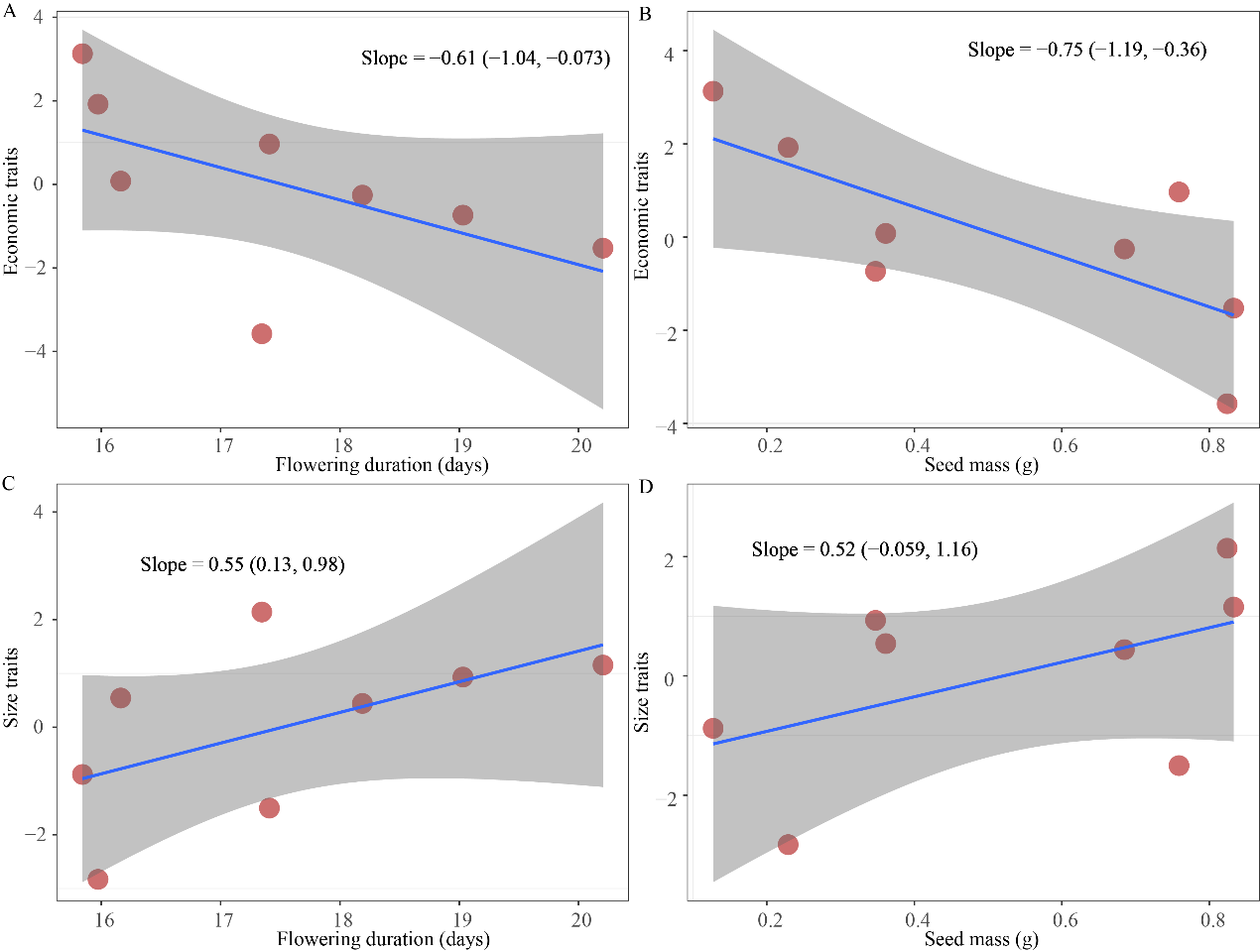


**Figure S17. The relationships between economic and size traits and flowering duration/seed mass based on the temporal plant community data.** Regression lines were fitted using the default ***lm()*** function in R, which employs ordinary least squares (OLS) regression, with shaded areas representing 95% confidence intervals. The slopes indicate the magnitude and direction of the linear associations between traits and response variables. Statistical significance of the slopes was evaluated using the bootstrap method with 999 permutations, providing robust estimates of uncertainty and reliability. The significance test of the slope was performed using the bootstrap method with 999 permutations.

**References:**

1.

Chen, Q., Wang, S., Borer, E.T., Bakker, J.D., Seabloom, E.W., Harpole, W.S. *et al.* (2023). Multidimensional responses of grassland stability to eutrophication. *Nature Communications*, 14, 6375.

2.

Guerrieri, R., Belmecheri, S., Ollinger, S.V., Asbjornsen, H., Jennings, K., Xiao, J. *et al.* (2019). Disentangling the role of photosynthesis and stomatal conductance on rising forest water-use efficiency. *Proceedings of the National Academy of Sciences*, 116, 16909-16914.

3.

He, N., Liu, C., Piao, S., Sack, L., Xu, L., Luo, Y. *et al.* (2019). Ecosystem Traits Linking Functional Traits to Macroecology. *Trends in Ecology & Evolution*, 34, 200-210.

4.

Isbell, F., Craven, D., Connolly, J., Loreau, M., Schmid, B., Beierkuhnlein, C. *et al.* (2015). Biodiversity increases the resistance of ecosystem productivity to climate extremes. *Nature*, 526, 574-577.

5.

Liu, S., García-Palacios, P., Tedersoo, L., Guirado, E., van der Heijden, M.G., Wagg, C. *et al.* (2022). Phylotype diversity within soil fungal functional groups drives ecosystem stability. 6, 900-909.

6.

Liu, Z., Dong, N., Zhang, H., Zhao, M., Ren, T., Liu, C. *et al.* (2021). Divergent long- and short-term responses to environmental gradients in specific leaf area of grassland species. *Ecological Indicators*, 130, 108058.

7.

Maxwell, T.M., Silva, L.C. & Horwath, W.R. (2018). Integrating effects of species composition and soil properties to predict shifts in montane forest carbon–water relations. *Proceedings of the National Academy of Sciences*, 115, E4219-E4226.

8.

Wang, J. & Wen, X. (2022). Excess radiation exacerbates drought stress impacts on canopy conductance along aridity gradients. *Biogeosciences*, 19, 4197-4208.

9.

Yan, P., He, N., Yu, K., Xu, L. & Van Meerbeek, K. (2023). Integrating multiple plant functional traits to predict ecosystem productivity. *Communications Biology*, 6, 239.
